# Comparative Effects of Crude Extracts and Bioactive Compounds from *Bidens pilosa* and *Bidens alba* on Nonspecific Immune Responses and Antibacterial Activity Against *Vibrio* sp. in Coculture with Lactic Acid Bacteria in Hybrid Grouper (*Epinephelus fuscoguttatus* ♀ × *Epinephelus lanceolatus* ♂)

**DOI:** 10.3390/ani14202990

**Published:** 2024-10-16

**Authors:** Ari Widodo, Huai-Ting Huang, Novi Rosmala Dewi, Bo-Ying Chen, Yu-Sheng Wu, Yeh-Fang Hu, Fan-Hua Nan

**Affiliations:** 1 Department of Aquaculture, National Taiwan Ocean University, Keelung City 20224, Taiwan; ariwidodo216@gmail.com (A.W.); twinkleqazwsx784@gmail.com (H.-T.H.); novi.rosmala.d@gmail.com (N.R.D.); joey860812@gmail.com (B.-Y.C.); yehfanghu@email.ntou.edu.tw (Y.-F.H.); 2 Department of Aquaculture, National Pingtung University of Science and Technology, Pingtung City 900, Taiwan; wuys0313@mail.npust.edu.tw

**Keywords:** hybrid grouper, medicinal plant, nonspecific immune response, antimicrobial activity, time-kill studies

## Abstract

This study investigated the effect of substances on nonspecific immune responses of head kidney leukocytes, the antimicrobial activity against *Vibrio* sp., as well as the time-kill of *Vibrio* sp. by combining the substances with lactic acid bacteria (LAB) and *Pediococcus* sp. The substances are *B. pilosa* hot water extract, *B. pilosa* powder extract, *B. pilosa* methanol extract, *B. pilosa* ethanol extract, *B. alba* hot water extract, *B. alba* powder extract, *B. alba* methanol extract, *B. alba* ethanol extract, and bioactive compounds, namely cytopiloyne, flavonoid, phenol, ethyl caffeate, luteolin, chlorogenic acid, butein, and linoleic acid. The results showed that some of them were nontoxic to the head kidney leukocytes, which can increase the phagocytic rate, phagocytic index, and respiratory burst. These substances were able to inhibit the growth of *Vibrio* sp.; they can even completely kill the pathogenic bacteria. The largest of the inhibition zone formed from the EC group at a concentration range of 5–50 µg/mL against *V. parahaemolyticus*, *V. alginolyticus*, and *V. harveyi* with a value of 19.7 ± 0.56, 19.3 ± 1.53, and 20.6 ± 1.53 mm. Furthermore, the time-kill studies showed that the LAB and *P. acidilactici* can completely kill the *Vibrio* sp. at 6 h incubation time, mainly in the group of combination with EC.

## Simple Summary

Intensive mariculture practices, particularly high stocking densities, have led to suboptimal growth rates and increased susceptibility to diseases in hybrid grouper, a popular species in aquaculture. Therefore, the use of medicinal plants has become promising to increase the immune response and resist disease. We examined the antimicrobial activity against Vibrio sp., because the *Vibrio* sp. is a common pathogen in fish. This study demonstrated that ethyl caffeate is nontoxic and increases the phagocytic rate, phagocytic index, and respiratory burst of head kidney leukocytes in hybrid grouper. Ethyl caffeate also has potent antimicrobial activity against some pathogenic bacteria in hybrid grouper aquaculture. Ethyl caffeate (EC) is considered a sustainable way to address the current challenges as they are cheap and environmentally friendly. Ultimately, it has potential to increase the metabolite secondary activity of beneficial bacteria.

## 1. Introduction

The Food and Agriculture Organization is committed to developing the fishery and aquaculture sectors as part of the Blue Transformation initiative [1]. This initiative focuses on the sustainable expansion and intensification of aquaculture production. Grouper is one of the species that needs to be highlighted as part of efforts to maintain a commitment to the Blue Transformation. Approximately 93% of global grouper production is concentrated in three countries and regions, China, Taiwan, and Indonesia, which accounts for 65%, 17%, and 11% of the total production, respectively [2]. Hybrid grouper farming yields a high income, has a favorable benefit–cost ratio, and offers a favorable profit margin due to the high market demand, rapid growth rate, high selling price, and short production cycle of the grouper [3]. However, the intensification of aquaculture has led to poor water quality, resulting in disease outbreaks. Vibriosis, a disease that causes substantial economic losses, is highly prevalent in grouper farms. This disease is caused by bacteria from the *Vibrio* genus, including *Vibrio vulnificus*, *Vibrio alginolyticus*, *Vibrio parahaemolyticus*, *Vibrio harveyi*, and *Vibrio anguillarum* [4]. To date, the primary methods for controlling vibriosis have been prevention and chemotherapeutic use. Fish farmers frequently administer antibiotics and disinfectants to treat infected fish. However, this practice does not align with the principles of the Blue Transformation. The misuse of medications has led to the accumulation of harmful antibiotic residues in fish and poses the risk that antibiotic-resistant bacteria will emerge in aquaculture systems. Thus, the use of medicinal plants to enhance the health of fish and shrimp has become increasingly popular.

Plant extracts contain various bioactive compounds, such as alkaloids, terpenoids, tannins, saponins, glycosides, flavonoids, phenolics, steroids, and essential oils [5]. These compounds can enhance fish health through multiple modes of action, such as by improving the innate immune response, acting as a natural antibiotic, promoting growth through improved feed utilization, and enhancing nutrient digestibility [6]. *Bidens pilosa*—commonly known as black-jack, beggar’s stick, or Spanish needle—belongs to the Asteraceae family [7]. This plant contains numerous bioactive compounds, including centaureidin, centaurein, luteolin (LT), butein (BT), linoleic acid (LA), ethyl caffeate (EC), chlorogenic acid (CA), and cytopiloyne (CP) [8,9,10,11,12]. A species in the same genus, *Bidens alba*, is also recognized as a medicinal plant and contains several bioactive compounds, such as CP, various phenolics, flavonoid (FV), steroids, tannins, and caffeic acid [13,14]. BT, FV, and LT, all classified as flavonoids, have been shown to have potential in various therapeutic areas, including preventing functional β-cell damage, exerting anti-HIV effects in mice, and improving the rate of survival of channel catfish against *Aeromonas hydrophila* [13,15]. LA, a polyunsaturated fatty acid, enhanced growth and lipid metabolism in coho salmon (*Oncorhynchus kisutch*) [16]. CP, a polyacetylenic glucoside, regulated T-cell differentiation and inhibited the development of nonobese diabetes (NOD) in NOD mice when used at the optimal dosage of 3 µg/mL [17]. CA, EC, and phenol (PH), which are natural phenolic compounds, can stimulate macrophages through the calcineurin pathway in human cells, exhibit antioxidant activity in juvenile largemouth bass (*Micropterus salmoides*), increase lipid peroxidation, and modulate catalase activity [13,18,19]. However, the specific modes of action of these compounds in cultured hybrid grouper fish have not yet been investigated. 

Currently, interest in traditional medicine is growing, and the need for additional therapeutics derived from plants is increasing. The surge in the popularity of medicinal plants is largely due to the belief that natural remedies are safer and more reliable than costly chemical drugs, which often have undesirable side effects [20]. Using plant-based treatments could address the escalating problem of resistance associated with conventional commercial antibiotics. Moreover, this approach could contribute to achieving the Blue Transformation in hybrid grouper farming. This study investigated the nonspecific immune responses of hybrid grouper enriched with two Bidens species (*B. pilosa* and *B. alba*), focusing on their specific bioactive compounds. Furthermore, their antimicrobial activities against fish pathogenic bacteria and the synergistic effects of beneficial bacteria were evaluated. By investigating these effects, the aim of this study is to identify the best candidate materials and dosages for future promising feed additives that can improve the health, disease resistance, and nontoxicity for both cells and the environment.

## 2. Materials and Methods

### 2.1. Hybrid Grouper Culture

Healthy hybrid grouper fish weighing 25–30 g was obtained from the Aquatic Animal Center at National Taiwan Ocean University (NTOU), Taiwan, for use in experiments. The fishes were acclimatized to laboratory conditions in composite tanks and fed a commercial diet twice daily at 3% of their body weight. Water exchange was performed daily at 25% of the tank volume. Water conditions were consistently controlled throughout the trial. The temperature ranged from 28 °C to 31 °C, salinity was maintained at 28 parts per thousand (ppt), and the dissolved oxygen level was maintained above 7 mg/L through continuous aeration. Ammonia and nitrate levels were maintained below 0.05 mg/L [21].

### 2.2. Material Preparation

The substances used in this study consisted of two types of herbal medicines: *B. pilosa* and *B. alba*. These plants were collected from a local garden at NTOU, Keelung, Taiwan. After collection, the whole plants were thoroughly cleaned using distilled water and dried at 50 °C for 48 h. The dried plants were ground into powder without any extraction and are referred to as *B. pilosa* powder (BP PW) and *B. alba* powder (BA PW). In addition, the dried herbs were extracted using hot water by following a previously reported protocol [22] with minor modifications. These herbs were also extracted using methanol and ethanol in accordance with a previously described method [23] with minor modifications. For the hot-water extraction, 100 g of ground material was added to 1000 mL of deionized water. The suspension was boiled for 30 min and then cooled to room temperature. For the methanol and ethanol extractions, the powders of *B. pilosa* and *B. alba* were each mixed with methanol or ethanol, respectively, in a 1:10 ratio and agitated at room temperature for 24 h. After boiling and incubation, each suspension was centrifuged at 3000× *g* for 10 min and filtered through a vacuum pump equipped with 0.22 µm pore-size filter paper (Whatman No. 2). The filtrates were lyophilized under reduced pressure by using a freeze-drying system to obtain dry extracts. The resulting extracts—labeled *B. pilosa* hot water extract (BP HW), *B. alba* hot water extract (BA HW), *B. pilosa* methanol extract (BP ME), *B. pilosa* ethanol extract (BP ET), *B. alba* methanol extract (BA ME), and *B. alba* ethanol extract (BA ET)—were stored at −20 °C prior to use.

The bioactive compounds used in this study were obtained commercially. CA (TCI, Tochigi, Japan), LT (TCI, Tochigi, Japan), LA (Cayman, East Ellsworth RD, Ann Arbor, MI, USA), CP (Sigma-Aldrich, Burlington, MA, USA), EC (Chem Faces, Wuhan, China), BT (TCI, Tochigi, Japan), PH (Sigma-Aldrich, Burlington, MA, USA), and FV (Cayman, East Ellsworth RD, Ann Arbor, MI, USA) were categorized as bioactive compounds of *B. pilosa* and *B. alba*. These compounds were dissolved in appropriate solvents to prepare stock solutions. CA, EC, FV, LA, LT, and PH were dissolved in dimethyl sulfoxide (DMSO). BT and CA were dissolved in methanol and hot water, respectively. Each compound was then diluted in Hank’s balanced salt solution (HBSS; Gibco, Waltham, MA, USA) to concentrations ranging from 0 to 500 µg/mL.

### 2.3. Cell Viability and Nonspecific Immune Responses of Head Kidney Leukocytes

#### 2.3.1. Head Kidney Leukocyte Extraction

Sixth individual (27 ± 0.3 g) were used for *in vitro* study in each material. The head kidney leukocytes were extracted from healthy hybrid grouper by using a previously described protocol [22] with minor modifications. Fish were randomly selected, anesthetized using MS-222 (10 mg/L; Sigma-Aldrich, Qingdao, China) for 10 min [24], and dissected at low temperature to carefully remove their head kidneys. The head kidneys were minced and rinsed with HBSS. The tissue was filtered through 100 μm nylon mesh (BD Falcon, Sigma-Aldrich, Burlington, MA, USA) and layered on 35% and 50% Percoll density gradients (GE Healthcare, Sigma-Aldrich, Burlington, MA, USA). The mixture was centrifuged at 400× *g* for 40 min at 4 °C. Subsequently, leukocytes were collected from the second layer and washed three times with HBSS. The total number of head kidney leukocytes was determined using a hemocytometer and counted under an inverted phase-contrast microscope (Leica DMIL, Leica Microsystems, Wetzlar GmbH, Wetzlar, Germany) in both the top and bottom fields (1 mm × 1 mm). The leukocyte concentration was adjusted to 2 × 10^6^ cells/mL with HBSS for further analysis. This extraction is quoted from Lee et al. 2020 [22]. 

#### 2.3.2. Cell Viability

The viability of the head kidney leukocytes was assessed using the 3-(4,5-dimethylthiazol-2-yl) 2,5-diphenyl tetrazolium bromide (MTT) assay as described previously [25] with minor modifications. The leukocyte solution was pipetted into a 96-well plate, and the adhered leukocytes were incubated with various concentrations of the substances in triplicate at room temperature for 30 min. HBSS served as the negative control. After incubation, the material solution was removed, and 100 µL of MTT solution (0.5 mg/mL in HBSS) was added to each well. The plate was then incubated for 4 h at room temperature in the dark to allow formazan crystal formation. Subsequently, 100 µL of DMSO was added to each well to dissolve the formazan crystals [26]. The plate was shaken for 10 min, and optical density was measured at 570 nm by using an enzyme-linked immunoassay (ELISA) reader. Cell viability was calculated using the following equation:

Cell viability (OD) = (OD of exposed cells)/(OD of control cells) × 100

#### 2.3.3. Phagocytic Activity

The phagocytic activity of head kidney leukocytes was measured by following a previously reported method [27] with minor modifications. Initially, 100 μL of leukocytes was placed on a cover glass and incubated for 60 min to allow cell adherence. The cells were then incubated with various concentrations of the substances for 30 min prior to analysis. After incubation, the leukocytes were washed with HBSS and then exposed to 100 μL of latex beads (0.8 μm; 3 × 10^7^ beads/mL, Sigma-Aldrich) for 60 min at room temperature. Following another wash with HBSS, the leukocytes were fixed with methanol for 5 min, stained with 6% Giemsa for 20 min, rinsed with distilled water, air-dried, and observed under a light microscope (Olympus, Shinjuku City, Japan). The phagocytic cells in 200 leukocytes were counted with a total of three fields of view at a magnification of 20×. Phagocytic activity was defined as the phagocytic rate (PR) and phagocytic index (PI), and both were calculated using the following equations:

PR (%) = (Phagocytic cell)/(Total leukocytes) × 100 PI = (Beads in phagocytic cell)/(Total phagocytic cell)

#### 2.3.4. Respiratory Burst

The respiratory burst of the head kidney leukocytes was measured using a previously described method [28]. The analysis was based on the reduction of nitro blue tetrazolium (NBT) to formazan, serving as an indicator of respiratory burst generation. Initially, the leukocytes were incubated with various concentrations of the substances for 30 min prior to analysis. Then, 100 μL of zymosan (1 mg/mL, Sigma-Aldrich) or 100 μL of HBSS was added to a 96-well plate, and incubation was performed for 30 min at room temperature to measure total and background superoxide (O_2_^−^) production, respectively. Zymosan (0.1 mg/mL, HBSS; Sigma-Aldrich) was added to stimulate the leukocytes. Following this incubation, the supernatant was discarded, and the leukocytes were incubated with 100 μL of NBT solution (0.3%) for 30 min at room temperature. To stop the reaction, the NBT solution was removed, and 100 μL of methanol was added to each well. After 5 min, the methanol was discarded. The wells were then washed three times with 70% methanol (100 μL each time) and air-dried for 30 min. The insoluble formazan crystals formed were dissolved by adding 120 μL of 2 M KOH and 140 μL of DMSO. The optical density was measured at 630 nm by using an ELISA microplate reader (Sigma-Aldrich, Burlington, MA, USA). Respiratory burst production was calculated using the following equation:

O_2_^−^ production = (OD of sample − OD of background)/(OD of background) × 100

### 2.4. Antibacterial Activity Against Vibrio sp.

#### 2.4.1. Bacterial Preparation

This study used bacteria retrieved from glycerol stocks stored at −80 °C in the Aquaculture and Physiology Laboratory, NTOU, Keelung, Taiwan. The bacteria were a *Vibrio* sp. group consisting of *V. alginolyticus*, *V. parahaemolyticus*, and *V. harveyi* and a lactic acid bacteria (LAB) group consisting of *L. reuteri*, *L. plantarum*, *L. acidophilus*, and *Pediococcus acidilactici*. Initially, all bacteria were cultured from the stock in tryptic soy broth (TSB; Merck, Germany) supplemented with 2% NaCl and were incubated at 28 °C for 24 h. Media for marine species are typically supplemented with 1% to 2% NaCl [29]. Subsequently, each bacterium was streaked on specific media: thiosulfate citrate bile salt sucrose (TCBS; Merck) for *Vibrio* sp. and De Man, Rogosa, and Sharpe (MRS; Neogen, USA) agar for LAB. These were incubated at 28 °C for ± 24 h. Isolated bacteria on agar were then used as working stocks for the *in vitro* study.

#### 2.4.2. Measurement of Bacterial Growth

The standard growth of each bacterium was measured prior to further analysis. Bacterial growth was measured at 0, 6, 12, 18, and 24 h by using the total plate count method, as described previously [30]. In this method, the spread plate technique was used. Each bacterium was initially cultured in TSB supplemented with 2% NaCl for *Vibrio* sp. and MRS broth for LAB. Serial dilutions were prepared in each corresponding medium for each bacterium. Then, a micropipette was used to transfer 100 µL from the last dilution to a Petri dish containing the specific medium for each bacterium. The bacterial sample was spread evenly by using a Drigalsky rod under aseptic conditions near a Bunsen burner. The plates were then incubated for 24 h, after which bacterial colonies growing on the media were counted using a colony counter. The standard bacterial culture growth was used to adjust the initial density of bacteria required for this study. The number of colony-forming units (CFU) per milliliter was calculated using the following formula:

CFU/mL = 1/(Volume × Dilution Factor) × Total colonies of bacteria

#### 2.4.3. Minimum Inhibitory Concentration and Bactericidal Concentration Tests

The minimum inhibitory concentration (MIC) and minimum bactericidal concentration (MBC) were determined using the broth dilution method, as described previously [31]. First, 1 mL of each crude extract and bioactive compound was prepared by dissolving them in TSB (for three *Vibrio* species) and MRS broth (for four LAB species) at various concentrations. Then, 10 µL of each bacterium (*V. alginolyticus*, *V. parahaemolyticus*, *V. harveyi*, *L. reuteri*, *L. plantarum*, *L. acidophilus*, and *P. acidilactici*, at 1 × 10^7^ CFU/mL) was cultured overnight and added to each tube containing different concentrations of the crude extracts and bioactive compounds. Two tubes containing 1 mL of TSB and MRS broth without bacterial inoculation served as negative controls. All tubes were incubated for 24 h at 28 °C. The lowest concentration of extracts and bioactive compounds that resulted in the smallest number of bacterial colonies on agar was considered the MIC, and the concentration that resulted in no visible colonies was considered the MBC. Each experiment was performed three times. 

#### 2.4.4. Inhibition Zone Test

The inhibition zone test was conducted using the two-layer agar method [32]. Initially, all media, including TSB semisolid containing 2% NaCl, were prepared. Bacteria were cultured overnight in the incubator. Sterile paper disks were impregnated with crude extracts and bioactive compounds at concentrations determined from the MIC and MBC test results. Subsequently, all bacteria at a final density of 10^5^ CFU/mL (based on the standard bacterial growth result) were separately inoculated in TSB semisolid and overlaid on TSA (for *Vibrio* sp.) or MRS (for LAB) agar medium. A 6 mm sterile disk (Advantec) impregnated with the extracts or compounds was placed on each agar plate and incubated at 28 °C for 24 h. Bleach solution was used as a positive control for sensitivity testing. Paper disks soaked with the corresponding solvents (DMSO, methanol, and hot water) were used as negative controls. The tests were performed in triplicate. Antibacterial activity was evaluated based on the diameter of the inhibition zones formed around the disks. 

### 2.5. Time-Kill Experiment in Coculture Against Some Vibrio sp.

A time-kill experiment was conducted using methods described previously [33,34]. Initially, principal component analysis (PCA) was performed using MATLAB software version 8 to determine the optimal dosages and substances. On the basis of the PCA results, the following experiments were conducted:

1. Experiment 1: *Vibrio* sp. at 10^5^ CFU/mL
2. Experiment 2: *Vibrio* sp. at 10^5^ CFU/mL + EC at 25 µg/mL
3. Experiment 3: *Vibrio* sp. at 10^5^ CFU/mL + CP at 25 µg/mL
4. Experiment 4: *Vibrio* sp. at 10^5^ CFU/mL + BP HW at 250 mg/L
5. Experiment 5: *Vibrio* sp. at 10^5^ CFU/mL + BP PW at 150 mg/L
6. Experiment 6: *Vibrio* sp. at 10^5^ CFU/mL + BA HW at 250 mg/L
7. Experiment 7: *Vibrio* sp. at 10^5^ CFU/mL + BA PW at 150 mg/L

Once the crude extracts and bioactive compounds had been added, bacterial inoculation was performed immediately. The flasks were then incubated at room temperature on an orbital shaker operated at 120 rpm for 48 h. Sampling was conducted at 0, 2, 6, 12, 24, 36, and 48 h. These samples were used to estimate the density of cocultured LAB and *Vibrio* sp. as well as to determine the timing of *Vibrio* sp. death due to the resistance conferred by the LAB enriched with the crude extracts and bioactive compounds. During each sampling, 1000 µL was taken and transferred to a microtube for subsequent dilutions. Samples from each dilution were cultured on TCBS and MRS agar to monitor the trends of growth in *Vibrio* sp. and LAB, respectively. *Vibrio* sp. and LAB were grown in each medium by using the drop plate method [35]. For this method, 20 µL of each sample was dropped on the agar, and this technique was performed three times per sample. The drops were allowed to dry, and the plates were incubated at 28 °C for 24–48 h. Bacterial density was then estimated by counting the colonies that developed at each drop point, and the results are expressed in CFU/mL.

### 2.6. Statistical Analysis

a. Analysis of Variance

The results of the cell viability, phagocytic activity, phagocytic index (PI), O_2_^−^ production, MIC, MBC, inhibition zone test, and time-kill experiments were compiled using MS Excel 2019 and are presented as the mean ± standard deviation from three replicates. Significant differences between groups were determined using one-way analysis of variance (ANOVA) followed by Tukey’s test. All statistical analyses were performed using IBM SPSS Statistics package 22.0 (SPSS Inc., Armonk, NY, USA). Differences were considered statistically significant at a *p* value of < 0.05. Additional ANOVA tests were conducted to examine significant differences in material dosages, and means were considered statistically significant at a *p* value of < 0.05.

b. PCA

PCA was performed to identify the optimal conditions for the substances used. Statistical analysis was performed using SAS (version 9.4), and diagrams, including heatmaps, were generated using Excel 2019. A *p* value of <0.05 indicated statistical significance. The mathematical expression for principal component 1 (PC1) is represented as follows:

“Prin1 = 0.415 cell viability + 0.522 phagocytic rate + 0.521 phagocytic index + 0.533 respiratory burst”.

## 3. Results

### 3.1. Effect of B. pilosa on Cell Viability, Phagocytic Activity, and Respiratory Burst

**Figure 1:**
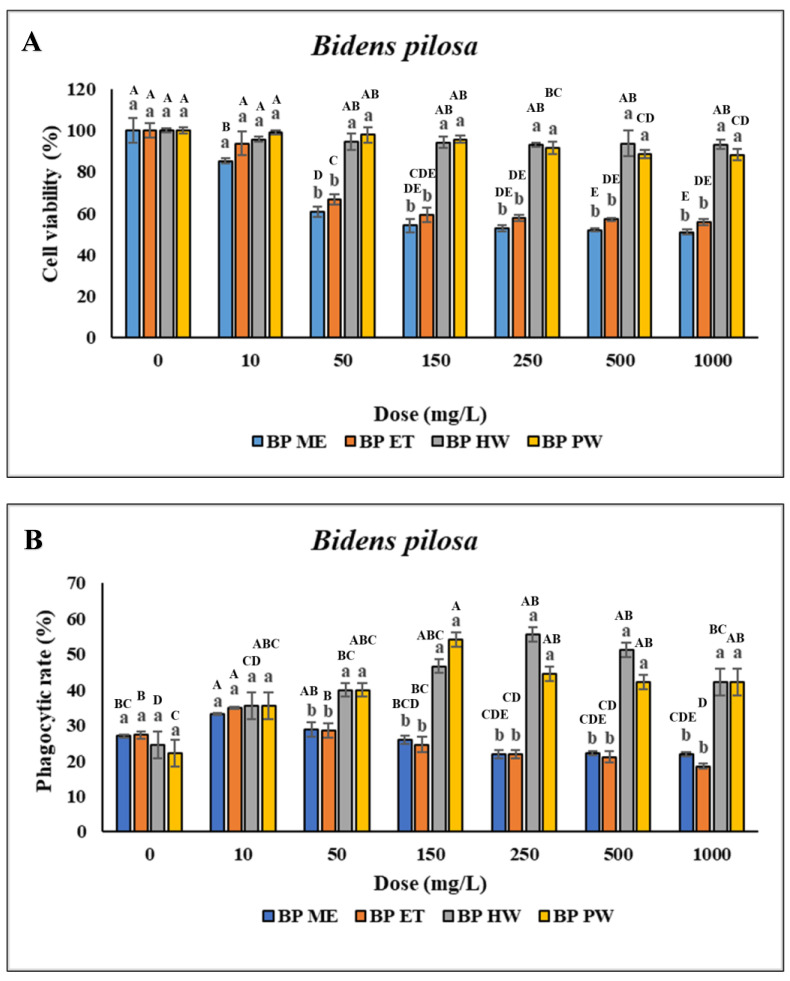

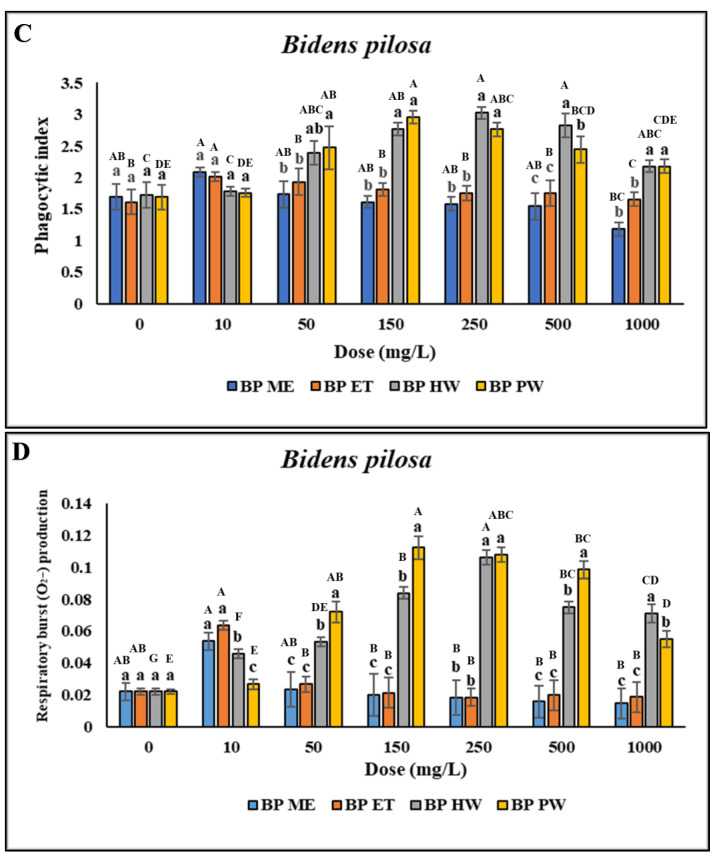
Nonspecific immune responses of leukocytes after incubation with different extraction of *B. pilosa*. (**A**) Cell viability; (**B**) Phagocytic rate; (**C**) Phagocytic index; (**D**) Respiratory burst production. Values are presented as the mean ± standard deviation for three replicates (n = 3). Significant differences (*p* < 0.05) between different extracts at the same concentration are indicated by lowercase letters above the bars. Statistically significant differences between different concentrations for the same extract are denoted by uppercase letters above the bars (*p* < 0.05).

No significant difference was found in the viability of the head kidney leukocytes treated with 10–1000 mg/L BP HW compared with the control (*p* > 0.05). However, the viability of the head kidney leukocytes treated with 2000–4000 mg/L BP HW was lower than that of the control (the data presented in this figure represents a subset of the complete dataset provided in Supplementary Materials). Furthermore, no significant difference in the viability of the head kidney leukocytes treated with 10–150 mg/L BP PW was found compared with the control (*p* > 0.05). However, the viability of the head kidney leukocytes treated with 250–4000 mg/L BP PW was lower compared with the control. Treatment of the head kidney leukocytes with 10 mg/L BP ME and BP ET did not significantly affect their viability. However, treatment of the head kidney leukocytes with 25–4000 mg/L BP ME and BP ET reduced their viability (Figure 1A). The PR of the head kidney leukocytes treated with 10 mg/L BP ME was significantly higher (*p* < 0.05) than that for the control. However, treatment of the leukocytes with 25–1000 mg/L BP ME did not significantly affect their PR (*p* > 0.05), and treatment with >2000 mg/L BP ME led to a gradual significant decrease in their PR (Figure 1B). The PI was unchanged across the various doses of BP ME and found to be significantly lower than for the control when used at concentrations > 2000 mg/L. Furthermore, the PR of the head kidney leukocytes significantly decreased (*p* < 0.05) after treatment with 10–25 mg/L BP ET but gradually decreased after treatment with 100–4000 mg/L BP ET. Similarly, the PI of the leukocytes significantly decreased after treatment with >100 mg/L BP ET. The PR and PI of the leukocytes was significantly higher (*p* < 0.05) following treatment with BP HW and BP PW compared with the control. The PR and PI of the leukocytes significantly increased (*p* < 0.05) for BP HW concentrations of 25 and 10 mg/L, respectively, peaking for 250 mg/L, but decreased as the concentration was increased to above 500 mg/L. The highest PR and PI were those for the leukocytes treated with 150 mg/L BP PW (*p* < 0.05); they were lower than this peak for BP PW concentrations above 250 mg/L (Figure 1C). O_2_^−^ production did not significantly differ (*p* > 0.05) between the different concentrations of BP ME and BP ET. However, treatment with 10–4000 mg/L BP HW resulted in a significant increase in O_2_^−^ production (*p* < 0.05), with the highest increase discovered for a concentration of 250 mg/L. Moreover, O_2_^−^ production was significantly increased (*p* < 0.05) following treatment with 25–4000 mg/L BP PW, with the highest value for a concentration of 150 mg/L (Figure 1D). Overall, the highest immune activity was observed in the cells with BP HW and BP PW.

### 3.2. Effect of B. alba on Cell Viability, Phagocytic Activity, and Respiratory Burst

**Figure 2:**
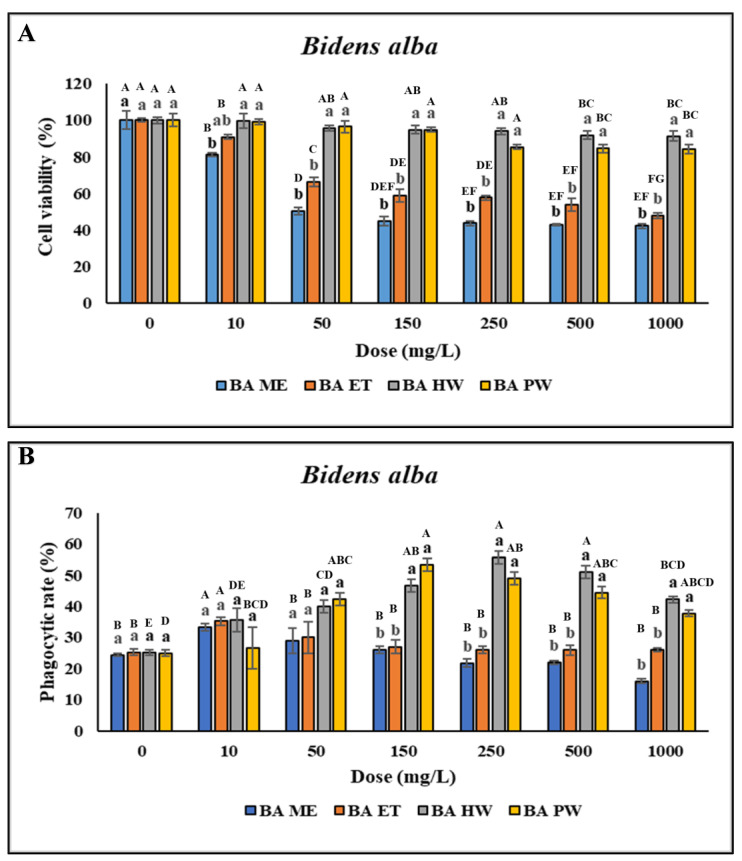

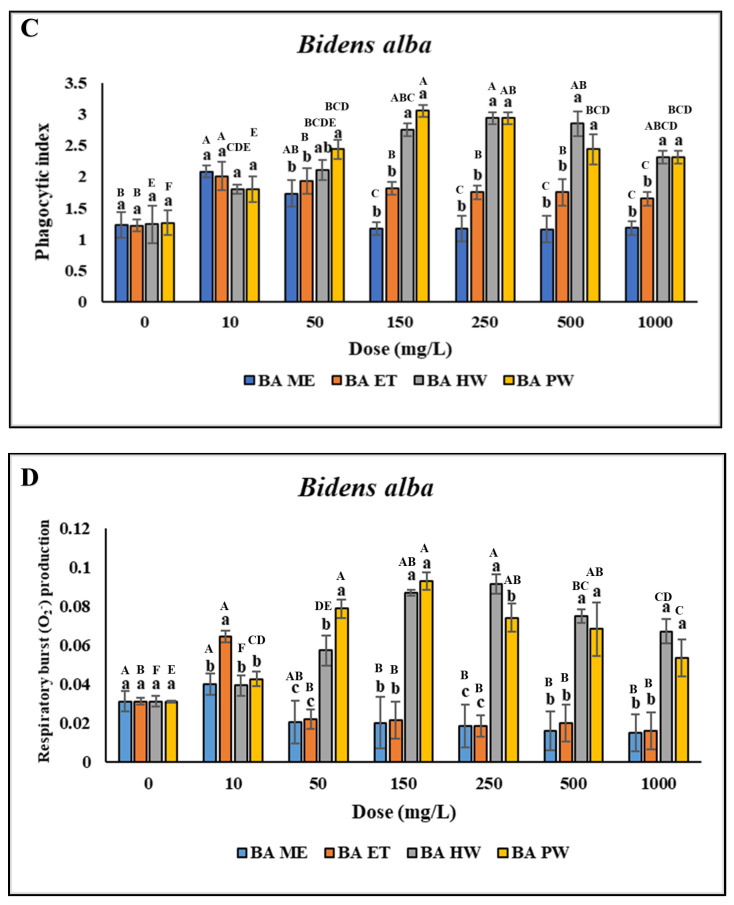
Nonspecific immune responses of leukocytes after incubation with different extraction of *B. alba*. (**A**) Cell viability; (**B**) Phagocytic rate; (**C**) Phagocytic index; (**D**) Respiratory burst production. Values are presented as the mean ± standard deviation for three replicates (n = 3). Significant differences (*p* < 0.05) between different extracts at the same concentration are indicated by lowercase letters above the bars. Statistically significant differences between various concentrations for the same extract are denoted by uppercase letters above the bar (*p* < 0.05).

No significant difference was discovered in the viability of the head kidney leukocytes treated with 10–250 mg/L BA HW and BA PW compared with the control (*p* > 0.05). However, cell viability was lower after treatment with 500–4000 mg/L BA HW or BA PW, as indicated by uppercase letters above the bars for the same extract treatments in Figure 2A. Furthermore, viability was lower after treatment with 10–4000 mg/L BA ME or BA ET (Figure 2A). This study determined significant increases in the PR and PI following treatment with 10–25 mg/L BA ME and BA ET and only 10 mg/L BA ME; however, the PR and PI were not so high when the concentrations were above 50 mg/L. With an increase in the BA ET concentration, the PR and PI of the leukocytes significantly increased (*p* < 0.05; concentrations of 10–25 mg/L) and then decreased (concentrations > 50 mg/L). These findings indicated that the PR and PI of the leukocytes treated with BA HW or BA PW were significantly higher than for the control (*p* < 0.05). With an increase in the BA HW concentration, the PR and PI of the treated leukocytes increased (concentrations of 10–250 mg/L), peaked (concentration of 250 mg/L; *p* < 0.05), and then decreased (concentrations > 500 mg/L). The highest PR and PI of leukocytes treated with BA PW were those for a concentration of 150 mg/L (*p* < 0.05). The PR and PI of the leukocytes treated with BA PW decreased as the concentration was increased above 250 mg/L (Figure 2B,C). No significant difference was found in O_2_^−^ production (*p* > 0.05) at BA ME concentrations ranging from 10 to 100 mg/L. However, O_2_^−^ production exhibited a gradual significant decrease (*p* < 0.05) as the BA ME concentration was increased above 150 mg/L compared with the control. Similarly, for BA ET, no significant differences were discovered in O_2_^−^ production across all concentrations (*p* > 0.05). However, a significant increase (*p* < 0.05) in O_2_^−^ production after treatment with 50–2000 mg/L BA HW was found, with the highest value observed at a concentration of 250 mg/L (Figure 2D). Overall, these findings demonstrated that the cells treated with BA HW or BA PW had the highest PR and PI.

### 3.3. Effect of Bioactive Compounds on Cell Viability, Phagocytic Activity, and Respiratory Burst

**Figure 3:**
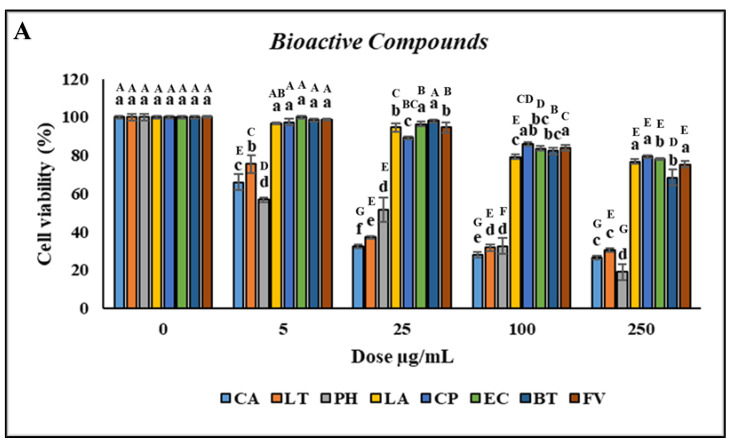

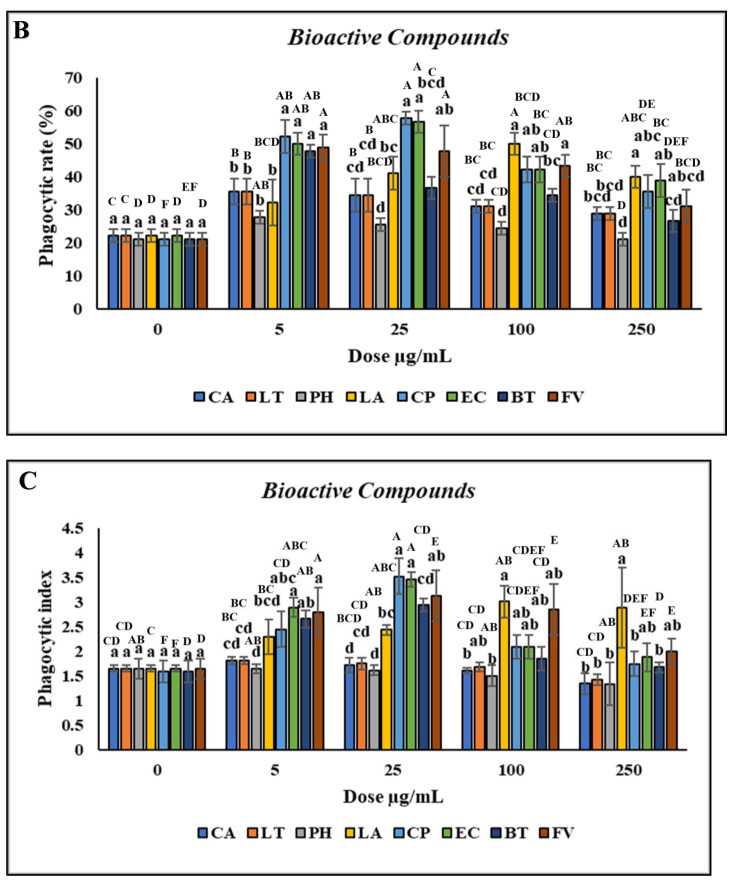

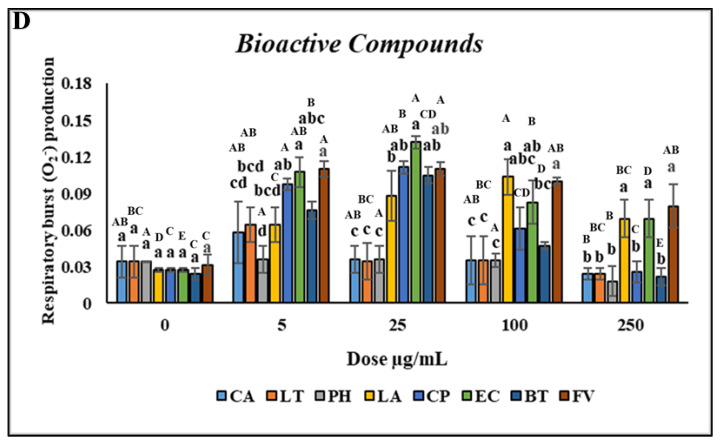
Nonspecific immune responses of leukocytes after incubation with various concentrations of bioactive compounds. (**A**) Cell viability; (**B**) Phagocytic rate; (**C**) Phagocytic index; (**D**) Respiratory burst production. Values are presented as the mean ± standard deviation for three replicates (n = 3). Significant differences (*p* < 0.05) between different bioactive compounds at the same concentration are indicated by lowercase letters above the bars. Statistically significant differences between various concentrations for the same bioactive compound are denoted by uppercase letters above the bars (*p* < 0.05).

Treatment with CA and LT at concentrations of 0.5–500 µg/mL significantly reduced cell viability. However, treatment with PH did not affect cell viability when the concentration was 0.5 µg/mL but decreased it when the concentration was 1–500 µg/mL compared with the control, as indicated by the uppercase letters in Figure 3A. No significant difference in the viability of the head kidney leukocytes was observed (*p* > 0.05) after treatment with 0.5–5 µg/mL LA and CP. However, treatment with 10–500 µg/mL LA and CP reduced cell viability. Furthermore, no significant difference in the viability of the head kidney leukocytes was discovered (*p* > 0.05) after treatment with 0.5–10 µg/mL EC and FV. However, treatment with 25–500 µg/mL EC and FV reduced cell viability. Similarly, no significant difference in cell viability was observed following treatment with 0.5–25 µg/mL BT. However, treatment with 50–500 µg/mL BT reduced cell viability (Figure 3A). As the concentrations of CA and LT were increased, the PR and PI significantly increased (*p* < 0.05; concentrations of 0.5–1 µg/mL) and then decreased (concentrations > 2.5 µg/mL).

No significant differences (*p* > 0.05) in the PR or PI were discovered between the leukocytes treated with PH versus those treated with the control. However, the PR and PI significantly increased (*p* < 0.05) after treatment with 10–250 µg/mL and 100–250 µg/mL LA, respectively, with the highest increase noted at a concentration of 150 µg/mL. Furthermore, the PR and PI significantly increased (*p* < 0.05) after treatment with 5–50 µg/mL and 1–250 µg/mL CP, respectively, with the highest increase noted at a concentration of 25 µg/mL (Figure 3B,C). Similar to CP, the highest increases in the PR and PI after treatment with EC (*p* < 0.05) were for the concentration of 25 µg/mL. The PR and PI of the leukocytes significantly increased (*p* < 0.05) after treatment with 1–100 and 2.5–25 µg/mL BT, respectively, with the highest increase discovered for a concentration of 10 µg/mL. Furthermore, the PR and PI of the leukocytes significantly increased (*p* < 0.05) following treatment with 0.5–500 and 1–500 µg/mL FV, respectively (Figure 3D). Overall, CP and EC treatments resulted in the highest increases in the PR and PI of the leukocytes.

### 3.4. MIC, MBC, and Inhibition Zone Against Vibrio sp. and LAB

None of the crude extract substances, regardless of their concentration, were found to inhibit or kill the beneficial bacteria used in this study (Table 1). However, some substances were effective against the tested *Vibrio* sp. The MIC values for *Vibrio* sp. were 150 mg/L for BP PW; 250 mg/L for BP HW, BA HW, and BA PW; 500 mg/L for BP ME and BA ME; and 1000 mg/L for BA ET and BP ET. However, the BP ME, BP ET, BA ME, and BA ET extracts did not completely kill *Vibrio* sp. bacteria. By contrast, the BA PW, BA HW, BP HW, and BP PW could kill *Vibrio* sp. bacteria when used at concentrations of 1000, 2000, 1000 or 2000, and 1000 or 2000 mg/L, respectively. 

The bioactive compounds were more effective against pathogenic bacteria, as indicated by the MIC and MBC values (Table 2). However, the bioactive compounds also inhibited the growth of beneficial bacteria, indicating that these compounds could inhibit the growth of normal microbial inhabitants in hybrid grouper. CA, LT, and FV inhibited the growth of *Vibrio* sp. at low MIC values of 2.5, 0.5, and 50 µg/mL, respectively, but they also inhibited the growth of beneficial bacteria at similar MIC values. By contrast, higher concentrations of LA, BT, and PH were required to inhibit *Vibrio* sp., with MIC values of 150, 150, and 250 µg/mL, respectively. CP and EC were the most potent against *Vibrio* sp., with the lowest MIC values of 25 and 5 µg/mL, respectively. However, EC did not inhibit the growth of or kill beneficial bacteria. The variation in MIC and MBC values between Gram-negative and Gram-positive bacteria may be attributable to differences in their cell surface structures.

The antibacterial activities of all substances were examined using disk diffusion assays against both pathogenic and beneficial bacteria. The results are presented in Table 3. At concentrations of 250–500 mg/L, BP HW and BA HW formed inhibition zones against all *Vibrio* sp. Moreover, BP PW and BA PW formed inhibition zones starting at a concentration of 150 mg/L. Of the bioactive compounds, EC and CP exhibited strong bactericidal activity against all *Vibrio* sp. (Table 4), resulting in larger inhibition zones than other bioactive compounds did. However, these compounds did not inhibit any beneficial bacteria other than *P. acidilactici* (see Supplementary Materials). EC produced the largest inhibition zones against *V. parahaemolyticus*, *V. alginolyticus*, and *V. harveyi*, measuring 19.7 ± 0.56, 19.3 ± 1.53, and 20.6 ± 1.53 mm, respectively, for concentrations ranging from 5 to 50 µg/mL. By contrast, BP ET, BA ET, LT, and PH did not effectively inhibit bacteria, as evidenced by the lack of inhibition zones.

### 3.5. Time-Kill Experiments Performed in Coculture Against Some Vibrio sp.

**Figure 4:**
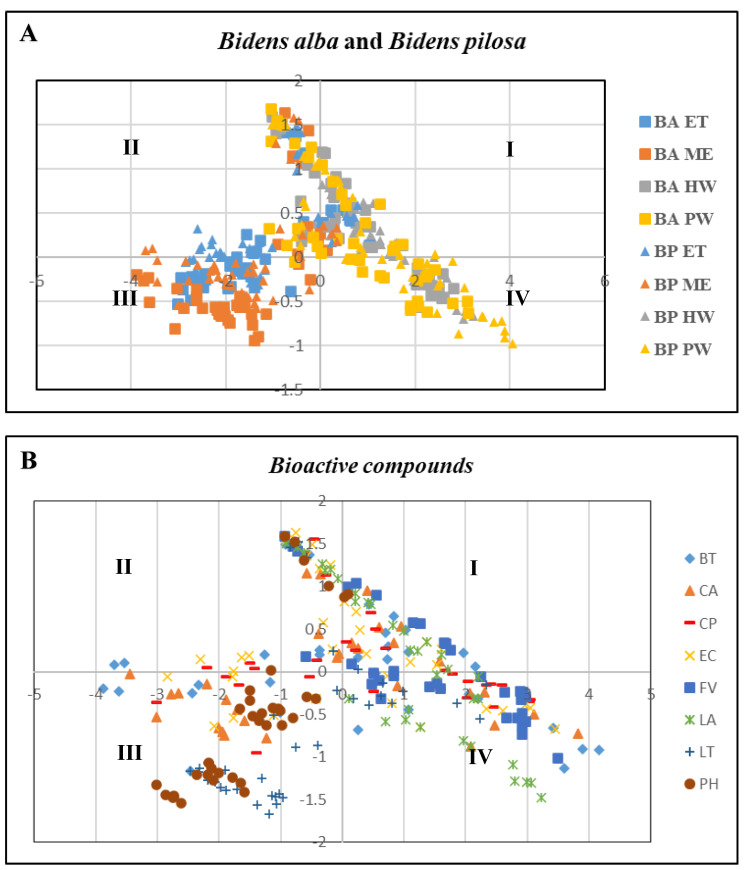
PCA of relationship between cell viability, phagocytic rate, phagocytic index, and respiratory burst. (**A**) Different substances from *B. pilosa* and *B. alba* extracts. (**B**) Different bioactive compounds. Arrows indicate the coefficients of correlation between the principal component scores and each material. Values are presented as the mean ± standard deviation for three replicates (n = 3) with significant differences (*p* < 0.05).

A time-kill experiment was performed to determine the synergistic effects of the substances and beneficial bacteria. The results obtained after the application of PCA to determine the optimal dosage and substances are depicted in Figure 4. The relationship among all the substances was analyzed based on values obtained for cell viability, PR, PI, and respiratory burst (Figure 4A,B) and categorized into four gradients (I, II, III, and IV). The results revealed that the first principal component (PC1) was the most critical because it indicated significant differences between the tested variables. 

EC was evenly distributed in Prin1, indicating that EC was the most effective material for enhancing cell viability, PR, PI, and respiratory burst. Furthermore, one-way ANOVA revealed that BP HW, BP PW, BA HW, BA PW, EC, and CP at concentrations of 250 mg/L, 150 mg/L, 250 mg/L, 150 mg/L, 25 µg/mL, and 25 µg/mL, respectively (Table 5 and Table 6), exhibited robust immunological and antibacterial properties. These findings were used in the subsequent time-kill experiment.

**Figure 5:**
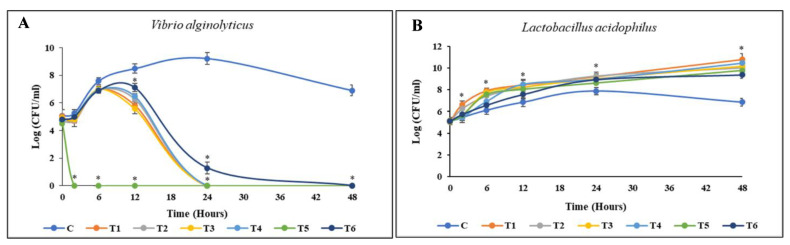

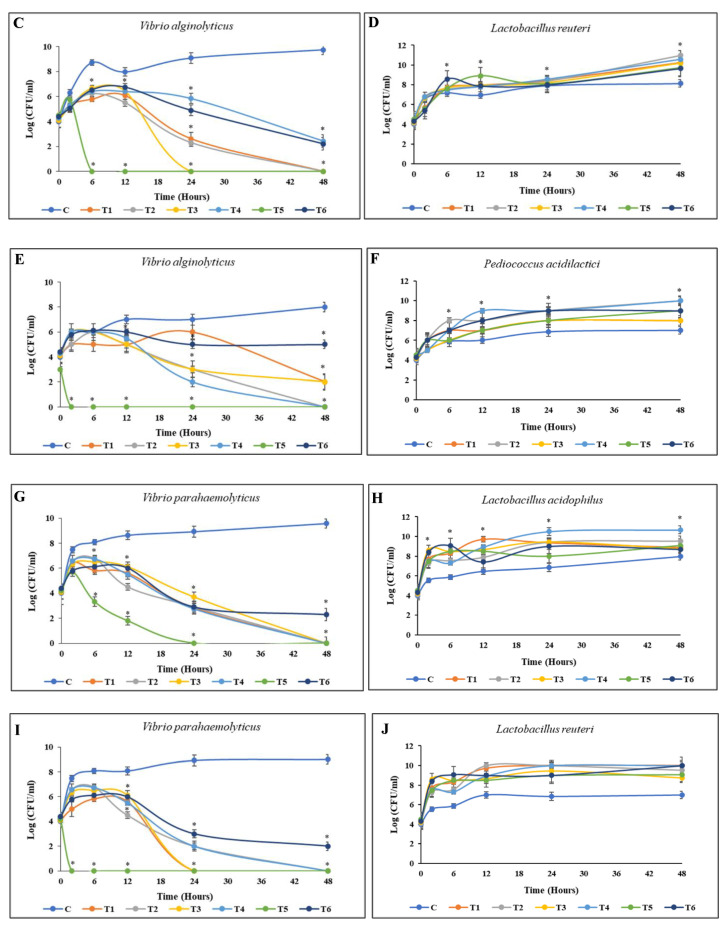

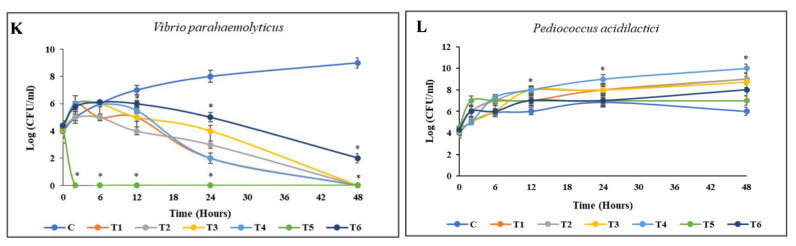
Time-kill curve activities of pathogenic bacteria. Cocultured between pathogenic bacteria and beneficial bacteria enriched with different materials in TSB saline medium. (**A**,**B**) *V. alginolyticus* with *L. acidophilus*; (**C**,**D**) *V. alginolyticus* with *L. reuteri*; (**E**,**F**) *V. alginolyticus* with *P. acidilactici*; (**G**,**H**) *V. parahaemolyticus* with *L. acidophilus*; (**I**,**J**) *V. parahaemolyticus* with *L. reuteri*; (**K**,**L**) *V. parahaemolyticus* with *P. acidilactici*. The initial density of both bacteria was 105 CFU/mL. Values are presented as the mean ± standard deviation for three replicates (n = 3). Significant differences (*p* < 0.05) between different extracts at each dosage level are indicated by an asterisk (*) above the bars. Control (**C**), coculture using 250 mg/L BP HW (T1), 150 mg/L BP PW (T2), 250 mg/L BA HW (T3), 150 mg/L BA PW (T4), 25 µg/mL EC (T5), and 25 µg/mL CP (T6).

The results of the coculture between *L. acidophilus* and *V. alginolyticus*, enriched with each material, are depicted in Figure 5. The entire population of *V. alginolyticus* was eliminated during the first 24 h of coculture incubation when enriched with BP HW, BP PW, BA HW, or BA PW. By contrast, in the EC enrichment group, *V. alginolyticus* was eliminated as early as 6 h. However, the growth of *L. acidophilus* remained stable, generally reaching a stationary phase. Similarly, against *L. reuteri* and *P. acidilactici*, the death of *V. alginolyticus* occurred before 24 h only in the EC enrichment group. However, BP PW, BP HW, and BA PW could all eliminate *V. alginolyticus* by 24 h without killing the beneficial bacteria. In a similar pattern, within 48 h of incubation, the entire population of *V. parahaemolyticus* was killed by *L. acidophilus* and *P. acidilactici* enriched with BP PW, BP HW, BA PW, or BA HW. However, EC enrichment killed *V. parahaemolyticus* before 24 h, with *L. reuteri* enriched EC achieving this as early as 6 h (Figure 5).

## 4. Discussion

*In vitro* cytotoxicity assays are commonly used to examine the toxicity of various inorganic and organic chemicals, including plant extracts and natural products. These assessments are aligned with international standards such as ISO 10993-5:2009 and are crucial for ensuring product safety, particularly the safety of products for human and animal cells. In this study, the MTT assay was performed to determine cell viability after exposure to the test substances. A tested material was considered cytotoxic when the cell viability had decreased to <70% when compared with the blank (control) [36]. The present results revealed that when cells were treated with BP HW, BP PW, BA HW, or BA PW at all tested concentrations, the cells’ viability remained above 80%, indicating that the extracts are safe for cells. However, for cells treated with BP ME, BP ET, BA ME, and BP ET, viability remained above 80% only when the extract concentrations were 10 to 25 mg/L. These results suggest that the materials that were extracted using methanol and ethanol solvent at high concentrations are harmful to cells or toxic. The BP ME, BP ET, BA ME, and BP ET, which contain a mixture of bioactive compounds at high concentrations, may negatively affect cell viability. A previous study indicated that inappropriate concentrations of bioactive compounds in a mixture can reduce the compounds’ biological effects [37] and may be unsafe for cells. Moreover, the present study investigated the viability of cells treated with bioactive compounds from *Bidens* sp. The viability of the cells treated with LA, CP, EC, or FV at concentrations ranging from 0.5 to 150 µg/mL was above 80%. In summary, the results revealed that the viability of the head kidney leukocytes treated with CA, PH, EC, FV, BT, LA, or CP was above 80% at 0.5 µg/mL, suggesting that these bioactive compounds are safe for head kidney leukocytes of hybrid grouper when used at low concentrations.

The effects of plant extracts on the immune system of hybrid grouper should be investigated before using them as ingredients in fish feed additive or drugs. This *in vitro* study examined phagocytic activity and respiratory burst production in head kidney leukocytes treated with all tested substances. The results revealed that all tested substances increased the PR, PI, and O_2_^−^ production in head kidney leukocytes in a dose-dependent manner. However, the optimum activity was found in the cells treated with BP HW, BP PW, BA HW, BA PW, EC, and CP. The optimal concentrations for increasing the nonspecific immune response of head kidney leukocytes were 150 mg/L for BP PW and BA PW, 250 mg/L for BP HW and BA HW, and 25 µg/mL for EC and CP. These results are in accordance with those of a previous study indicating that *B. pilosa* powder significantly increased reactive oxygen species production in cobia head kidney leukocytes when used at concentrations of 10, 20, 30, 40, 50, and 100 mg/mL [22]. In mice, CP enhanced phagocytosis and lysozyme activity [38] and functioned as a T-cell modulator when employed at the optimum concentration of 5 µg/mL [39]. Other studies have demonstrated that CA, flavonoids, and phenolic compounds exhibit antioxidant activities in various animals, such as the juvenile largemouth bass, zebrafish, and juvenile northern snakehead fish [18,19,40]. EC induced nitric oxide production in RAW 264.7 cells [41]. Consistent with these findings, the present results indicated that BP HW, BP PW, BA HW, BA PW, EC, and CP enhanced the nonspecific immune response of hybrid grouper *in vitro*.

The majority of probiotic bacteria in the intestines of hybrid grouper belong to the genus *Lactobacillus* sp. [20], including *L. acidophilus*, *L. plantarum*, and *L. reuteri*. This finding indicates that the natural intestinal microflora of hybrid grouper is rich in probiotic bacteria. This richness is strongly correlated with the pond culture environment and host organism itself. Thus, the use of prebiotics to preserve these naturally beneficial bacteria is a strategic approach to enhance the host’s defense against harmful bacteria. This study investigated the antimicrobial activities of various substances on the basis of MIC, MBC, inhibition zone tests, and time-kill experiments targeting *Vibrio* sp. in synergy with beneficial bacteria. The results of the MIC and MBC tests revealed that all substances could inhibit or kill *Vibrio* sp. bacteria at various concentrations. By contrast, at all tested concentrations, BP HW, BP PW, BA HW, BA PW, and EC did not inhibit the growth of or kill the beneficial bacteria used in this study. This finding was confirmed through the inhibition zone test. Similar to the MIC and MBC results, the inhibition zone test showed that BP HW, BP PW, BA HW, and BA PW produced inhibition zones against all *Vibrio* sp. when used at concentrations ranging from 150 to 500 mg/L. Moreover, EC and CP exhibited strong bactericidal activity against all *Vibrio* sp. but not LAB. A previous study identified several factors that lead to differences between the responses of *Vibrio* sp. and LAB to bioactive compounds, including differences in cell wall structure, secretion mechanisms, detoxification enzymes, and stress tolerance. *Lactobacillus* sp. is less susceptible to bioactive compounds than other species because of their strong cell membrane, efficient excretion system, effective detoxification enzymes, and high stress tolerance [42].

This study performed PCA and ANOVA to determine the optimum substances and dosages. The findings indicated that EC was the most effective compound for enhancing cell viability, PR, PI, and respiratory burst in hybrid grouper. A previous study demonstrated that at the optimal dosage of 1 µg/mL, EC markedly suppressed lipopolysaccharide-induced nitric oxide production [41]. Thus, a further analysis was conducted to evaluate the efficacy of this compound.

The time-kill assay results indicated that the combination of substances with LAB bacteria had a bactericidal effect on *Vibrio* sp. for various incubation durations. The combination of LAB and EC exerted a robust bactericidal effect, effectively killing *Vibrio* sp. within 6 h of incubation. This result indicates that EC may act as a prebiotic for beneficial bacteria, promoting their growth. However, the inhibitory action of *Lactobacillus* spp. on pathogenic bacteria is attributable to the generation of lactic acid and other organic acids, the reduction of enteroadhesion and aggregation by pathogenic bacteria, and the development of antimicrobial compounds, such as bacteriocins [43,44]. In particular, within a living organism, the presence of lactic acid and other organic acids produced by these probiotics and the formation of antimicrobial compounds, such as bacteriocins, directly inhibits the growth and proliferation of harmful microbes [42].

These results suggest that EC enhances the immune response of head kidney leukocytes in hybrid grouper and plays a crucial role in the defense mechanisms of diseased fish [45]. Moreover, EC was found to exhibit strong antibacterial activity against all *Vibrio* sp. and to have symbiotic properties with *Lactobacillus* sp., which could benefit hybrid grouper culture. The results of these experiments will provide a more comprehensive understanding of EC’s potential as the best candidate material as well as dosages for future promising feed additives for therapeutic agents for fish. Further experimental studies and *in vivo* experiments are needed to validate the efficacy and safety of EC in hybrid grouper.

## 5. Conclusions

This study demonstrated that treatment with BP HW, BP PW, BA HW, BA PW, CP, and EC maintained cell viability above 70% in a dose-dependent manner, indicating that these substances are safe for the head kidney leukocytes of hybrid grouper. Moreover, these compounds enhanced nonspecific immune responses and antimicrobial properties against *Vibrio* sp. Time-kill experiments indicated that these compounds promoted the growth of natural bacterial inhabitants, such as *Lactobacillus* sp., in hybrid grouper. In particular, EC exhibits strong antimicrobial properties and can act as a prebiotic candidate for hybrid grouper. Additional *in vivo* studies are needed to confirm these effects.

## Figures and Tables

**Table 1:** MIC and MBC against *Vibrio* sp., *Lactobacillus* sp., and *Pediococcus acidilactici*.

Crude Extract	*V. parahaemolyticus*		*V. alginolyticus*		*V. harveyi*		*L. plantarum*		*L. acidophilus*		*L. reuteri*		*P. acidilactici*	
	MIC (mg/L)	MBC (mg/L)	MIC (mg/L)	MBC (mg/L)	MIC (mg/L)	MBC (mg/L)	MIC (mg/L)	MBC (mg/L)	MIC (mg/L)	MBC (mg/L)	MIC (mg/L)	MBC (mg/L)	MIC (mg/L)	MBC (mg/L)
BP HW	250	2000	250	1000	250	1000	4000	>4000 *	4000	>4000 *	4000	>4000 *	4000	>4000 **
BP PW	150	2000	150	2000	150	1000	4000	>4000 *	4000	>4000 *	4000	>4000 *	4000	>4000 **
BP ME	500	>4000 ***	500	>4000 ***	500	>4000 ***	4000	>4000 *	4000	>4000 *	4000	>4000 *	4000	>4000 **
BP ET	1000	>4000 ***	1000	>4000 ***	1000	>4000 ***	4000	>4000 *	4000	>4000 *	4000	>4000 *	4000	>4000 **
BA HW	250	2000	250	2000	250	2000	4000	>4000 *	4000	>4000 *	4000	>4000 *	4000	>4000 **
BA PW	250	1000	250	1000	250	1000	4000	>4000 *	4000	>4000 *	4000	>4000 *	4000	>4000 **
BA ME	500	>4000 ***	500	>4000 ***	500	>4000 ***	4000	>4000 *	4000	>4000 *	4000	>4000 *	4000	>4000 **
BA ET	1000	>4000 ***	1000	>4000 ***	1000	>4000 ***	4000	>4000 *	4000	>4000 *	4000	>4000 *	4000	>4000 **

Values were derived from three parallel experiments. * *Lactobacillus* sp. colonies were still detected on MRS agar at a concentration of 4000 mg/L. ** *P. acidilactici* colonies were still detected on MRS agar at a concentration of 4000 mg/L. *** *Vibrio* sp. colonies were still detected on TCBS agar at a concentration of 4000 mg/L.

**Table 2:** MIC and MBC against *Vibrio* sp., *Lactobacillus* sp., and *Pediococcus acidilactici*.

Specific Bioactive Compounds	*V. parahaemolyticus*		*V. alginolyticus*		*V. harveyi*		*L. plantarum*		*L. acidophilus*		*L. reuteri*		*P. acidilactici*	
	MIC (µg/mL)	MBC (µg/mL)	MIC (µg/mL)	MBC (µg/mL)	MIC (µg/mL)	MBC (µg/mL)	MIC (µg/mL)	MBC (µg/mL)	MIC (µg/mL)	MBC (µg/mL)	MIC (µg/mL)	MBC (µg/mL)	MIC (µg/mL)	MBC (µg/mL)
CA	2.5	>500 ***	2.5	>500 ***	2.5	>500 ***	5	>500 *	5	>500 *	5	>500 *	5	>500 **
LT	0.5	>500 ***	0.5	>500 ***	0.5	>500 ***	0.5	>500 *	0.5	>500 *	0.5	>500 *	0.5	>500 **
LA	150	>500 ***	150	>500 ***	150	>500 ***	250	>500 *	250	>500 *	250	>500 *	500	>500 **
CP	25	250	25	250	25	250	250	>500 *	250	>500 *	250	>500 *	250	>500 **
EC	5	250	5	150	5	150	500	>500 *	500	>500 *	500	>500 *	50	>500 **
BT	250	>500 ***	150	>500 ***	150	>500 ***	500	>500 *	500	>500 *	500	>500 *	500	>500 **
PH	250	>500 ***	250	>500 ***	250	>500 ***	500	>500 *	500	>500 *	500	>500 *	500	>500 **
FV	50	>500 ***	50	>500 ***	50	>500 ***	100	>500 *	100	>500 *	100	>500 *	100	>500 **

Values were derived from three parallel experiments. * *Lactobacillus* sp. colonies were still detected on MRS agar at a concentration of 500 µg/mL. ** *P. acidilactici* colonies were still detected on MRS agar at a concentration of 500 µg/mL. *** *Vibrio* sp. colonies were still detected on TCBS agar at a concentration of 500 µg/mL.

**Table 3:** Inhibition zone of crude extracts against *Vibrio* sp., *Lactobacillus* sp., and *Pediococcus acidilactici*.

Extract	Dose (mg/L)	Inhibition Zone (mm)						
		*V. parahaemolyticus*	*V. alginolyticus*	*V. harveyi*	*L. plantarum*	*L. acidophilus*	*L. reuteri*	*P. acidilactici*
BP HW	100	NA	NA	NA	NA	NA	NA	NA
	150	NA	NA	NA	NA	NA	NA	NA
	250	7.4 ± 0.12 ^b^	7.53 ± 0.23 ^b^	7.40 ± 0.40 ^b^	NA	NA	NA	NA
	500	8.47 ± 0.12 ^a^	8.63 ± 0.06 ^a^	8.43 ± 0.25 ^a^	6.47 ± 0.06 ^a^	6.53 ± 0.12 ^a^	6.47 ± 0.12 ^a^	6.27 ± 0.12 ^a^
BP PW	100	NA	NA	NA	NA	NA	NA	NA
	150	6.87 ± 0.12 ^c^	7.07 ± 0.12 ^b^	7.0 ± 0.2 ^b^	NA	NA	NA	NA
	250	7.13 ± 0.12 ^b^	7.27 ± 0.12 ^b^	7.2 ± 0.2 ^b^	NA	NA	NA	NA
	500	7.73 ± 0.12 ^a^	7.87 ± 0.12 ^a^	7.8 ± 0.2 ^a^	6.27 ± 0.12 ^a^	6.33 ± 0.12 ^a^	6.27 ± 0.12 ^a^	6.47 ± 0.06 ^a^
BP ME	100	NA	NA	NA	NA	NA	NA	NA
	150	NA	NA	NA	NA	NA	NA	NA
	250	NA	NA	NA	NA	NA	NA	NA
	500	6.27 ± 0.12 ^a^	6.47 ± 0.12 ^a^	6.3 ± 0.1 ^a^	NA	NA	NA	NA
BP ET	100	NA	NA	NA	NA	NA	NA	NA
	150	NA	NA	NA	NA	NA	NA	NA
	250	NA	NA	NA	NA	NA	NA	NA
	500	NA	NA	NA	NA	NA	NA	NA
BA HW	100	NA	NA	NA	NA	NA	NA	NA
	150	NA	NA	NA	NA	NA	NA	NA
	250	7.53 ± 0.11 ^b^	7.61 ± 0.21 ^b^	7.46 ± 0.30 ^b^	NA	NA	NA	NA
	500	8.27 ± 0.12 ^a^	8.4 ± 0.1 ^a^	8.16 ± 0.21 ^a^	6.16 ± 0.05 ^a^	6.2 ± 0.1 ^a^	6.33 ± 0.05 ^a^	6.23 ± 0.05 ^a^
BA PW	100	NA	NA	NA	NA	NA	NA	NA
	150	6.97 ± 0.12 ^c^	7.12 ± 0.12 ^c^	7.1 ± 0.2 ^c^	NA	NA	NA	NA
	250	7.17 ± 0.05 ^b^	7.23 ± 0.06 ^b^	7.3 ± 0.1 ^b^	NA	NA	NA	NA
	500	7.56 ± 0.06 ^a^	7.57 ± 0.21 ^a^	7.86 ± 0.11 ^a^	6.23 ± 0.15 ^a^	6.37 ± 0.15 ^a^	6.3 ± 0.1 ^a^	6.43 ± 0.06 ^a^
BA ME	100	NA	NA	NA	NA	NA	NA	NA
	150	NA	NA	NA	NA	NA	NA	NA
	250	NA	NA	NA	NA	NA	NA	NA
	500	6.3 ± 0.1 ^a^	6.23 ± 0.15 ^a^	6.16 ± 0.05 ^a^	NA	NA	NA	NA
BA ET	100	NA	NA	NA	NA	NA	NA	NA
	150	NA	NA	NA	NA	NA	NA	NA
	250	NA	NA	NA	NA	NA	NA	NA
	500	NA	NA	NA	NA	NA	NA	NA

NA: No activity. Values are presented as the mean ± standard deviation for three replicates (n = 3). Significant differences (*p* < 0.05) between different extracts at each dosage level are indicated by lowercase letters above the bars.

**Table 4:** Inhibition zone of bioactive compounds against *Vibrio* sp., *Lactobacillus* sp., and *Pediococcus acidilactici*.

Specific Bioactive Compounds	Dose (µg/mL)	Inhibition Zone (mm)						
		*V. parahaemolyticus*	*V. alginolyticus*	*V. harveyi*	*L. plantarum*	*L. acidophilus*	*L. reuteri*	*P. acidilactici*
CA	0.5	NA	NA	NA	NA	NA	NA	NA
	1	NA	NA	NA	NA	NA	NA	NA
	2.5	6.5 ± 0.05 ^b^	6.6 ± 0.2 ^a^	7.40 ± 0.40 ^a^	NA	NA	NA	NA
	5	6.7 ± 0.06 ^a^	6.8 ± 0.2 ^a^	8.43 ± 0.25 ^a^	6.33 ± 0.57 ^a^	6.3 ± 0.57 ^a^	6.43 ± 0.2 ^a^	6.23 ± 0.057 ^a^
LT	0.5	NA	NA	NA	NA	NA	NA	NA
	1	NA	NA	NA	NA	NA	NA	NA
	2.5	NA	NA	NA	NA	NA	NA	NA
	5	NA	NA	NA	NA	NA	NA	NA
LA	50	NA	NA	NA	NA	NA	NA	NA
	100	NA	NA	NA	NA	NA	NA	NA
	150	6.5 ± 0.05 ^b^	6.5 ± 0.1 ^b^	6.56 ± 0.11 ^b^	NA	NA	NA	NA
	250	7.1 ± 0.1 ^a^	7.03 ± 0.06 ^a^	7.13 ± 0.05 ^a^	6.46 ± 0.05 ^a^	6.2 ± 0.1 ^a^	6.36 ± 0.57 ^a^	6.16 ± 0.05 ^a^
CP	10	10.3 ± 0.57 ^b^	9.6 ± 0.57 ^c^	10.6 ± 0.57 ^c^	NA	NA	NA	NA
	25	11 ± 1.2 ^b^	11 ± 1 ^bc^	11 ± 0.1 ^bc^	NA	NA	NA	6.5 ± 0.05 ^b^
	50	12 ± 1.1 ^b^	12.6 ± 0.6 ^b^	12.6 ± 1.57 ^b^	NA	NA	NA	7.4 ± 0.11 ^a^
	100	14.3 ± 0.57 ^a^	15 ± 1 ^a^	15.3 ± 0.57 ^a^	6.4 ± 0.2 ^a^	6.4 ± 0.2 ^a^	6.33 ± 0.11 ^a^	7.5 ± 0.12 ^a^
EC	5	12.3 ± 0.57 ^d^	13.3 ± 0.57 ^b^	13.1 ± 1.52 ^c^	NA	NA	NA	NA
	10	14.6 ± 0.57 ^c^	14.3 ± 1.15 ^b^	13.6 ± 1.52 ^bc^	NA	NA	NA	NA
	25	16.3 ± 0.56 ^b^	17.4 ± 0.57 ^a^	16.6 ± 0.57 ^b^	NA	NA	NA	7.5 ± 0.1 ^b^
	50	19.7 ± 0.56 ^a^	19.3 ± 1.53 ^a^	20.6 ± 1.53 ^a^	NA	NA	NA	8.5 ± 0.12 ^a^
BT	5	NA	NA	NA	NA	NA	NA	NA
	10	NA	NA	NA	NA	NA	NA	NA
	25	NA	NA	NA	NA	NA	NA	NA
	50	6.53 ± 0.057 ^a^	6.43 ± 0.15 ^a^	6.3 ± 0.11 ^a^	NA	NA	NA	NA
PH	5	NA	NA	NA	NA	NA	NA	NA
	10	NA	NA	NA	NA	NA	NA	NA
	25	NA	NA	NA	NA	NA	NA	NA
	50	NA	NA	NA	NA	NA	NA	NA
FV	5	NA	NA	NA	NA	NA	NA	NA
	10	NA	NA	NA	NA	NA	NA	NA
	25	6.5 ± 0.1 ^b^	6.46 ± 0.05 ^a^	6.73 ± 0.25 ^a^	NA	NA	NA	NA
	50	6.93 ± 0.11 ^a^	6.9 ± 0.3 ^a^	7.46 ± 0.55 ^a^	6.16 ± 0.05 ^a^	6.16 ± 0.05 ^a^	6.36 ± 0.05 ^a^	6.33 ± 0.15 ^a^

NA: No activity. Values are presented as the mean ± standard deviation for three replicates (n = 3). Significant differences (*p* < 0.05) between different extracts at each dosage level are indicated by lowercase letters above the bars.

**Table 5:** Best dose determination of crude extracts for cell viability and immunity by conducting ANOVA.

Crude Extract	Dose (mg/mL)										
	0	10	25	50	100	150	250	500	1000	2000	4000
BP ME	128.66978	120.66106	106.76661	91.275683	83.922963	81.700686	76.172239	75.632171	73.966302	70.286097	60.777215
BP ET	128.93089	130.58486	124.96977	96.980605	88.781692	85.796216	81.510072	79.943719	76.063114	72.075121	69.692979
BP HW	126.18867	133.17739	135.50306	136.98	141.28009	143.63015	151.962	147.7109	137.7453	130.75496	121.94723
BP PW	123.92978	136.49032	138.41595	140.42338	152.2565	152.82938	138.843	133.32008	132.58739	119.9654	112.85631
BA ME	125.62204	116.6499	104.31768	80.860153	74.276723	71.974831	66.704052	66.325812	59.368246	52.941725	44.29571
BA ET	126.55204	128.10515	123.60482	98.140778	91.199385	87.857121	85.361457	81.638062	75.51667	61.594941	55.646214
BA HW	126.43204	136.9656	137.64279	137.8765	141.58168	144.35332	152.57296	145.87678	136.01678	129.00605	117.58907
BA PW	126.25204	127.71872	133.52552	141.17868	146.74593	151.33947	137.27057	131.53269	124.33206	114.61101	102.38271

Values are presented as the mean ± standard deviation for three replicates (n = 3) with significant differences (*p* < 0.05). This analysis identified significant differences among dosages for various substances.

**Table 6:** Best dose determination of bioactive compounds for cell viability and immunity by conducting ANOVA.

Bioactive Compounds	Dose (µg/mL)											
	0	0.5	1	2.5	5	10	25	50	100	150	250	500
CA	123.90702	149.99011	134.02195	127.60824	103.47267	83.734107	68.658389	64.164423	60.593718	57.62961	56.967123	53.332773
LT	123.90702	140.7735	118.58029	115.0597	112.90018	90.501681	73.320501	68.671621	64.63929	61.63706	60.861669	59.295177
PH	122.78797	130.56853	98.807796	88.311693	86.355814	82.930432	78.858015	59.095338	58.590619	51.87813	41.504634	41.036064
LA	123.90035	130.612	130.79819	131.69414	131.05272	139.59844	138.18002	137.05689	132.39157	131.69355	119.68278	108.09936
CP	122.72575	132.39772	140.45046	140.381	150.18971	151.03944	152.58724	139.17952	130.45697	123.09137	116.70509	110.44937
EC	123.90035	134.33878	144.80795	143.69204	151.56082	152.47916	158.34149	144.69747	126.69281	116.23811	109.25791	104.11492
BT	122.72208	132.93386	139.90453	141.03611	149.07267	151.23616	136.79554	123.16716	118.63355	102.59078	96.782666	91.679372
FV	122.7853	134.46433	141.47831	144.68498	150.82606	150.99733	144.2822	140.29115	134.74191	120.52063	113.47133	108.11874

Values are presented as the mean ± standard deviation for three replicates (n = 3) with significant differences (*p* < 0.05). This analysis identified significant differences among dosages for various substances.

## Data Availability

The authors confirm that the data supporting the findings of this study are available within the article.

## Supplementary Materials

The following supporting information can be downloaded at: https://www.mdpi.com/article/10.3390/ani14202990/s1, Figure S1: Nonspecific immune responses of leukocytes after incubation with different extraction of *B. pilosa*; Figure S2: Nonspecific immune responses of leukocytes after incubation with different extractions of *B. alba*; Figure S3: Nonspecific immune responses of leukocytes after incubation with various concentrations of bioactive compounds; Figure S4: Representative figure of inhibition zones test from ethyl caffeate (EC) against some bacteria; Figure S5: The difference between *Bidens pilosa* and *Bidens alba* can be identified based on the hairs on its leaves.
