# Supplementary material for: Comparative Effects of Crude Extracts and Bioactive Compounds from Bidens pilosa and Bidens alba on Nonspecific Immune Responses and Antibacterial Activity Against Vibrio sp. in Coculture with Lactic Acid Bacteria in Hybrid Grouper (Epinephelus fuscoguttatus ♀ × Epinephelus lanceolatus ♂)
Source: Animals (Basel). 2024 Oct 16;14(20):2990. doi: 10.3390/ani14202990 (PMC11506134; doi:10.3390/ani14202990)
Supplement: Supplementary file 1 [file animals-14-02990-s001.zip › animals-3239352-supplementary file S1.pdf]

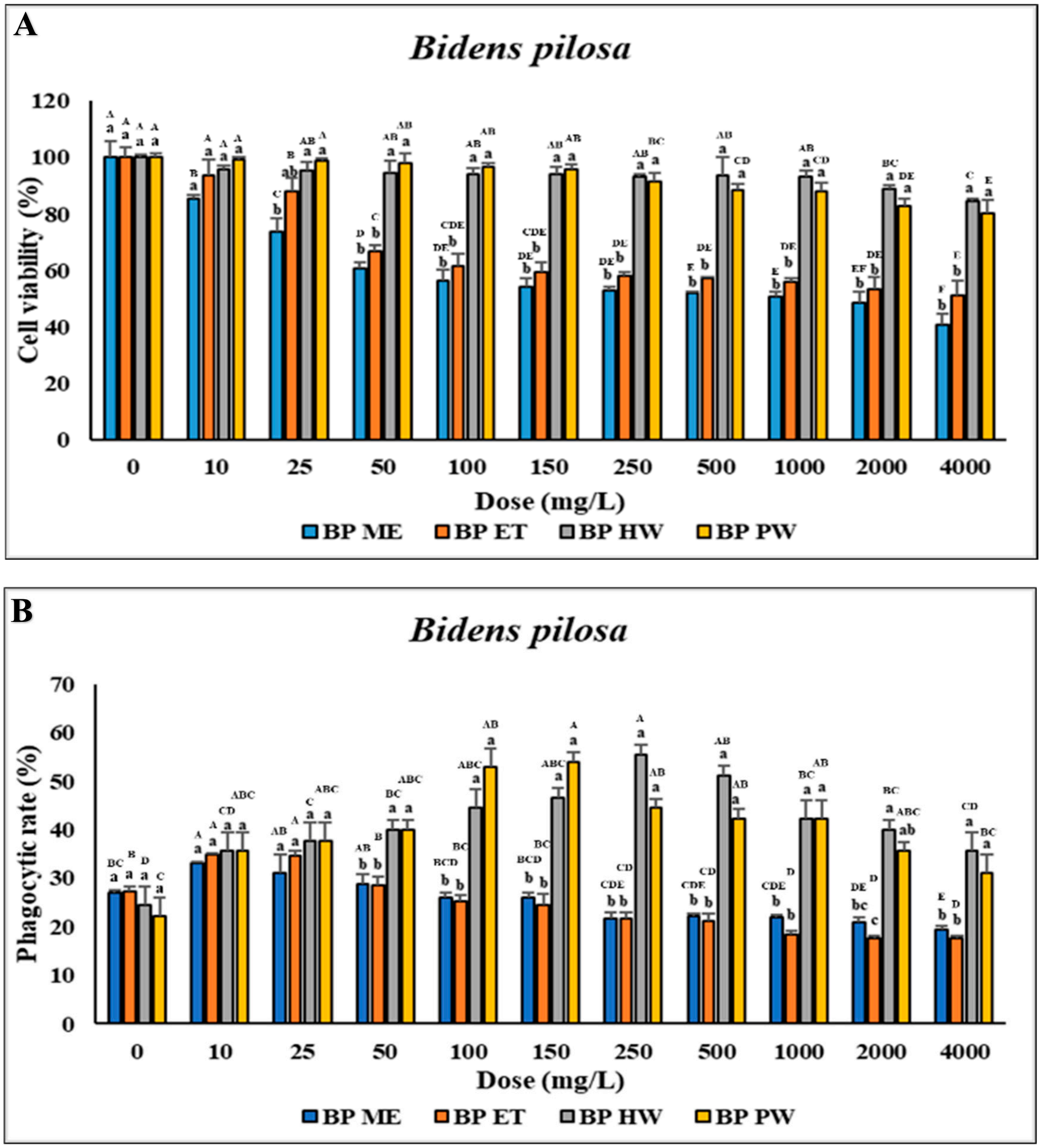
A
Bidens pilosa
Cell viability (%)
120
100
80
60
40
20
0
0
10
25
50
100
150
250
500
1000
2000
4000
Dose (mg/L)
BP ME
BP ET
BP HW
BP PW
B
Bidens pilosa
Phagocytic rate (%)
70
60
50
40
30
20
10
0
0
10
25
50
100
150
250
500
1000
2000
4000
Dose (mg/L)
BP ME
BP ET
BP HW
BP PW

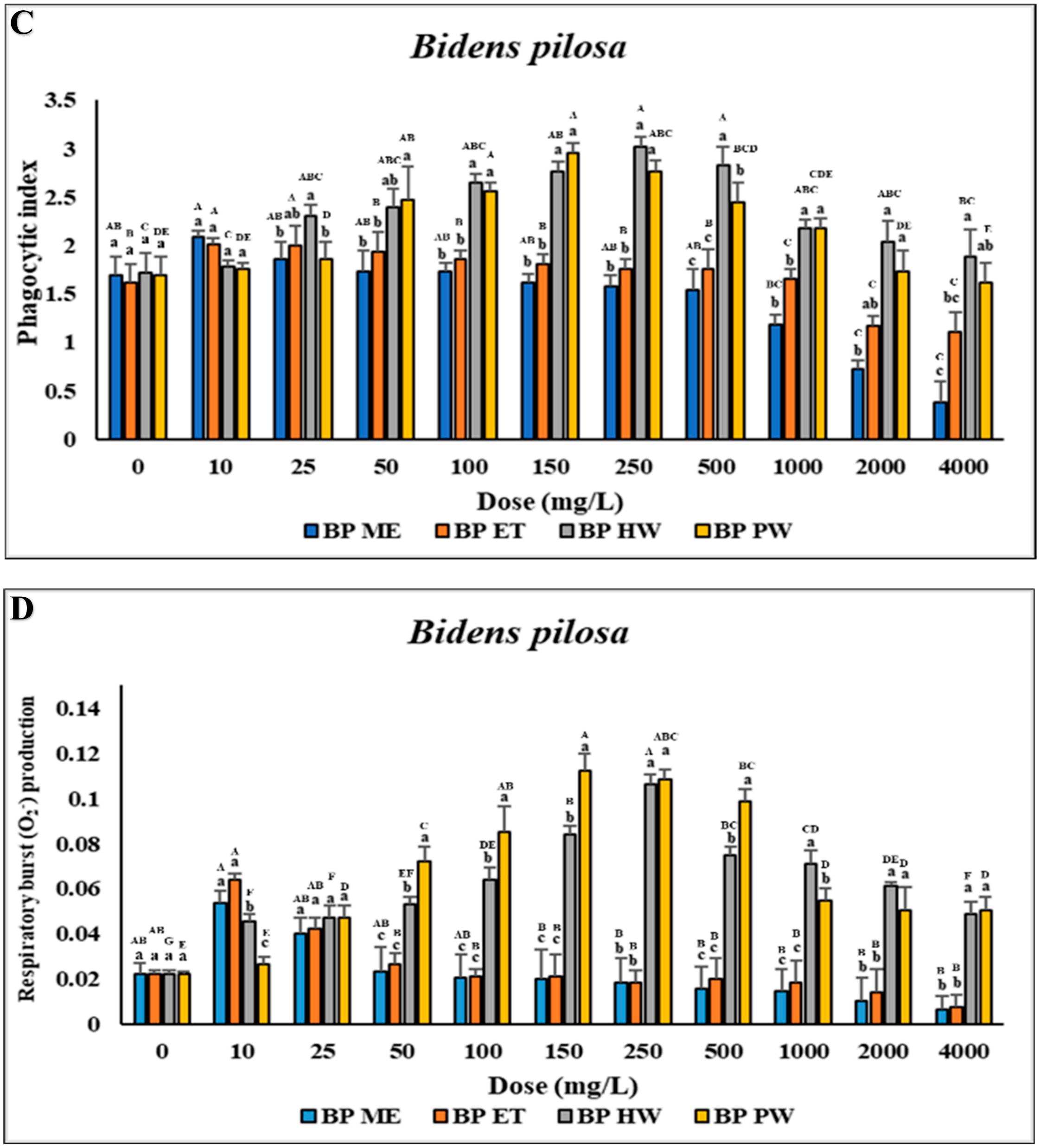

Figure S1. Nonspecific immune responses of leukocytes after incubation with different extraction of *B. pilosa*. Values are presented as the mean ± standard deviation for three replicates (n = 3). Significant differences ($p < 0.05$) between different extracts at the same concentration are indicated by lowercase letters above the bars. Statistically significant differences between different concentrations for the same extract are denoted by uppercase letters above the bars ($p < 0.05$).

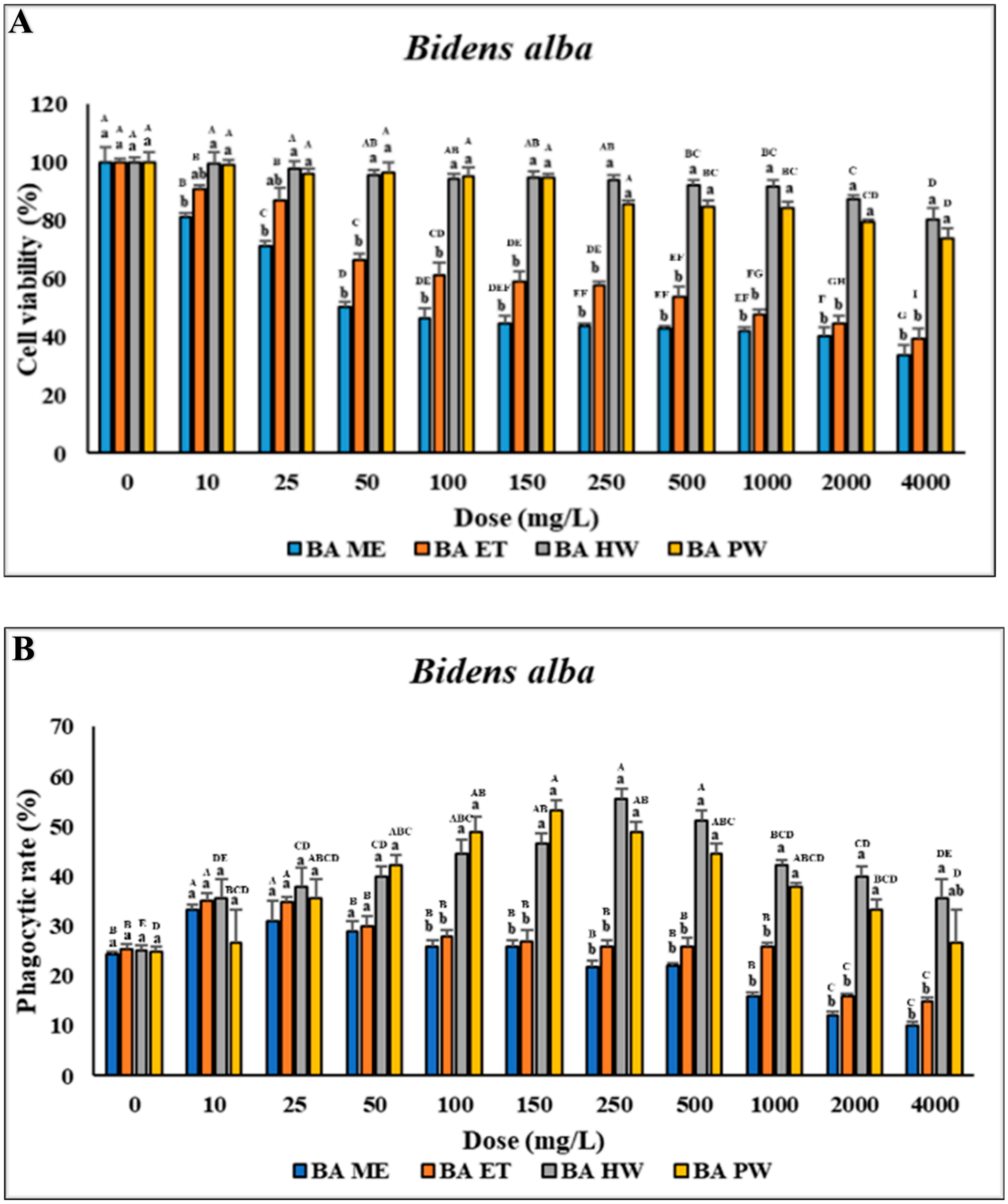
A
Bidens alba
Cell viability (%)
120
100
80
60
40
20
0
0
10
25
50
100
150
250
500
1000
2000
4000
Dose (mg/L)
BA ME
BA ET
BA HW
BA PW
B
Bidens alba
Phagocytic rate (%)
70
60
50
40
30
20
10
0
0
10
25
50
100
150
250
500
1000
2000
4000
Dose (mg/L)
BA ME
BA ET
BA HW
BA PW

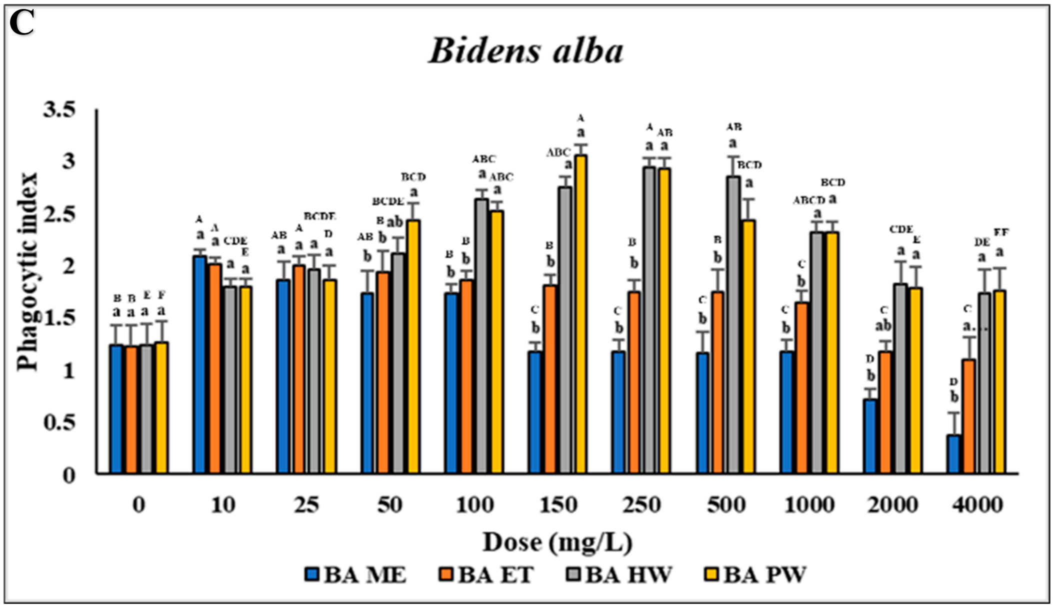

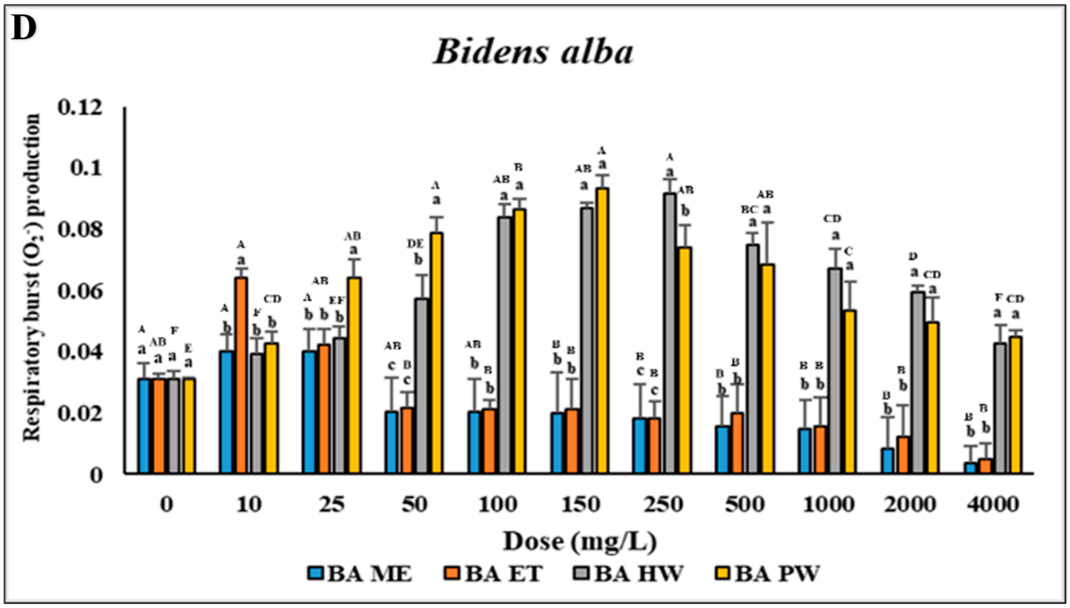

Figure S2. Nonspecific immune responses of leukocytes after incubation with different extraction of *B. alba*. Values are presented as the mean ± standard deviation for three replicates (n = 3). Significant differences ($p < 0.05$) between different extracts at the same concentration are indicated by lowercase letters above the bars. Statistically significant differences between various concentrations for the same extract are denoted by uppercase letters above the bar ($p < 0.05$).

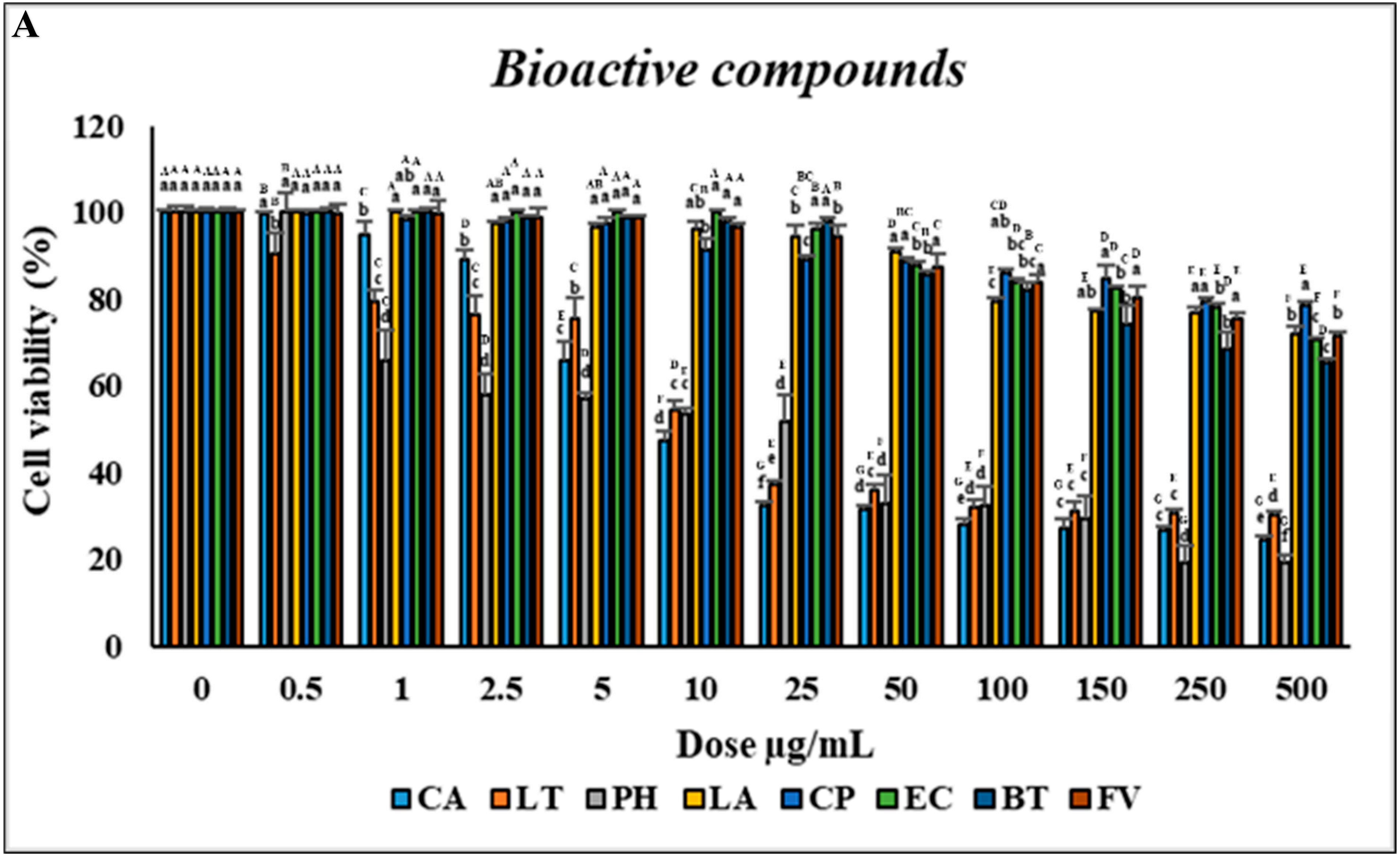
A
Bioactive compounds
Cell viability (%)
120
100
80
60
40
20
0
0
0.5
1
2.5
5
10
25
50
100
150
250
500
Dose µg/mL
CA
LT
PH
LA
CP
EC
BT
FV

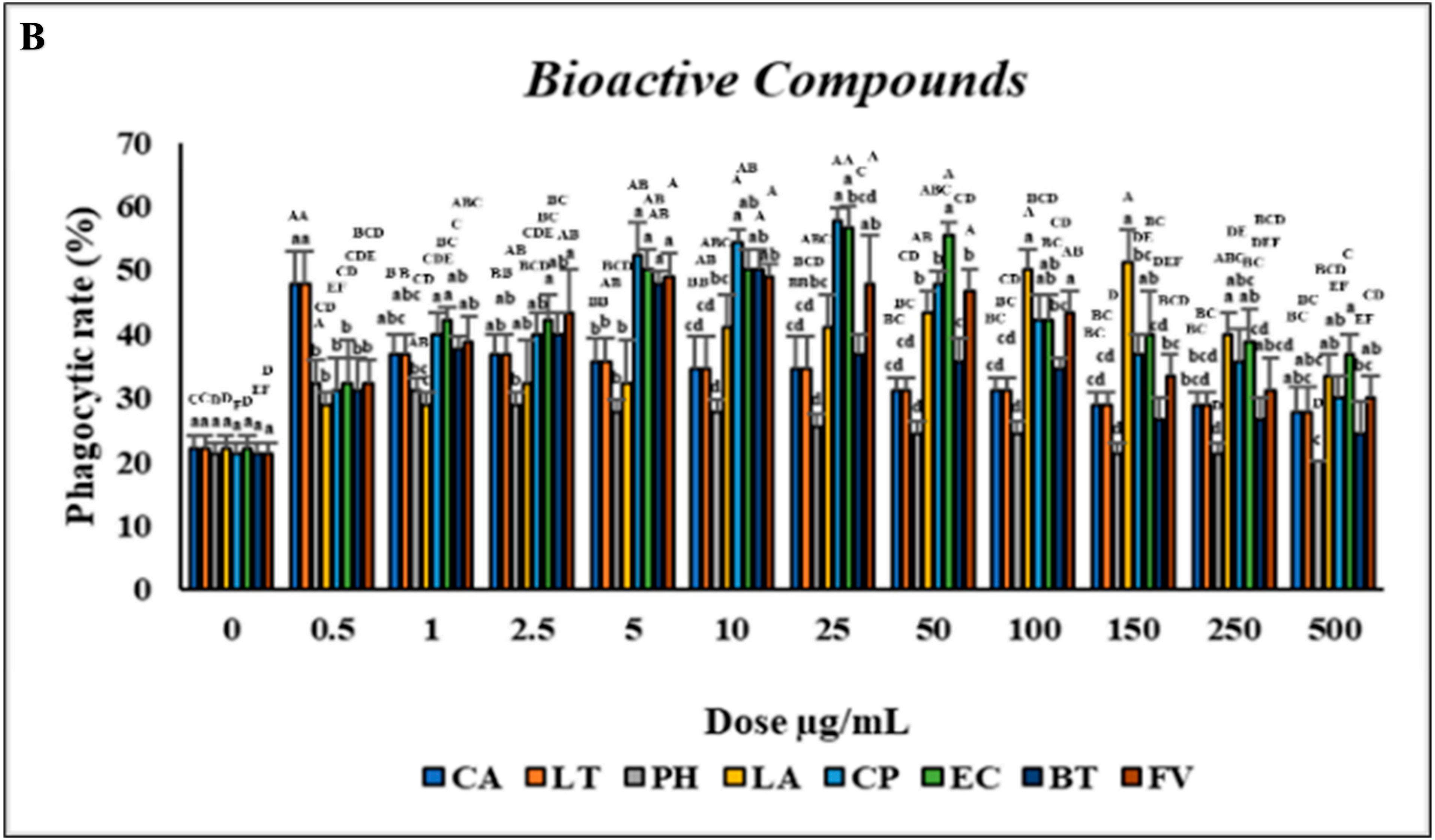

B
Bioactive Compounds
Phagocytic rate (%)
Dose µg/mL
0
0.5
1
2.5
5
10
25
50
100
150
250
500
CA
LT
PH
LA
CP
EC
BT
FV

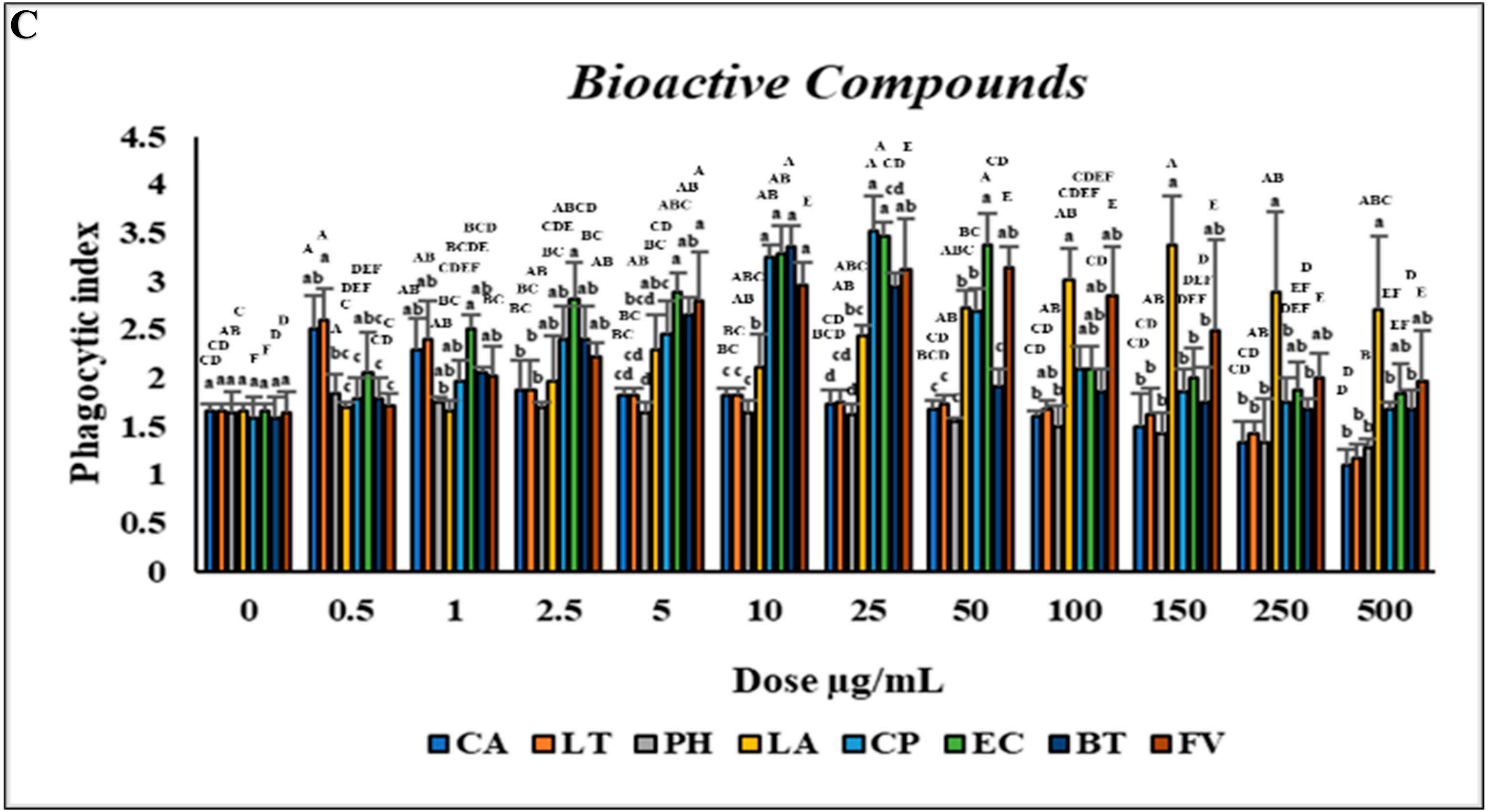
C
Bioactive Compounds
Phagocytic index
Dose µg/mL
CA LT PH LA CP EC BT FV
0
0.5
1
2.5
5
10
25
50
100
150
250
500

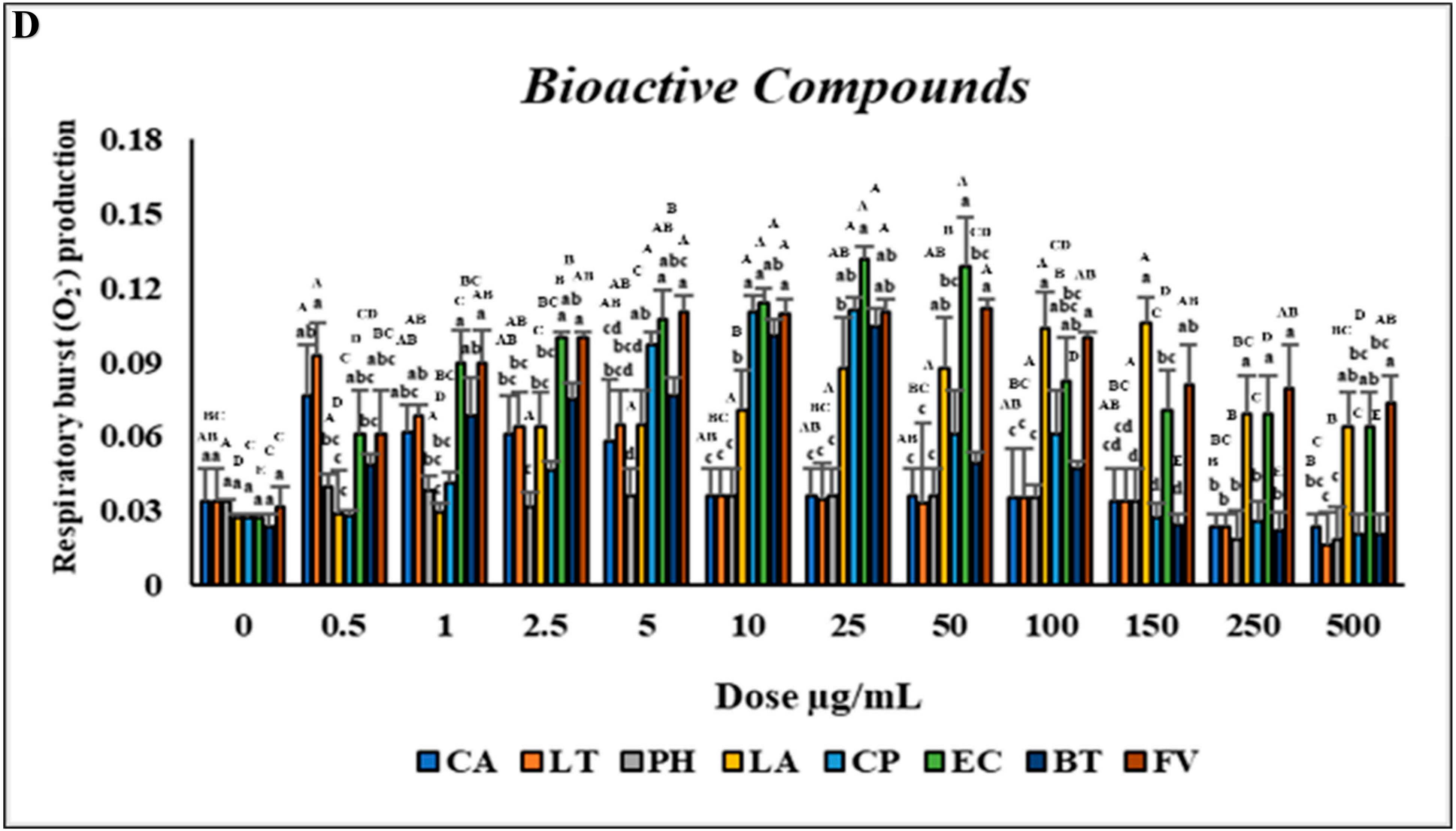
D
Bioactive Compounds
Respiratory burst (O2·-) production
0.18
0.15
0.12
0.09
0.06
0.03
0
0
0.5
1
2.5
5
10
25
50
100
150
250
500
Dose µg/mL
CA
LT
PH
LA
CP
EC
BT
FV

Figure S3. Nonspecific immune responses of leukocytes after incubation with various concentrations of bioactive compounds. Values are presented as the mean ± standard deviation for three replicates (n = 3). Significant differences ($p < 0.05$) between different bioactive compounds at the same concentration are indicated by lowercase letters above the bars. Statistically significant differences between various concentrations for the same bioactive compound are denoted by uppercase letters above the bars ($p < 0.05$).

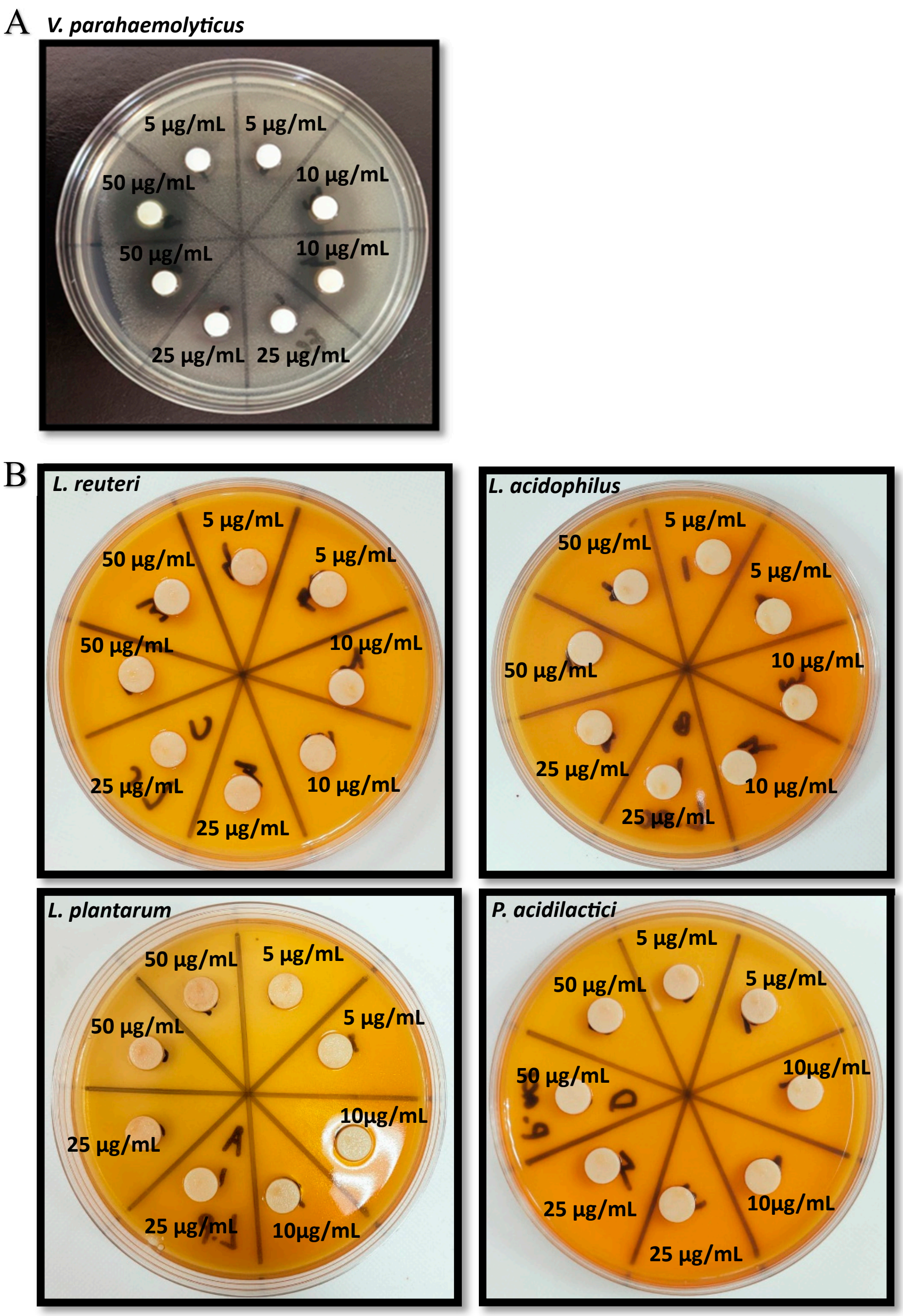

Figure S4. Representative figure of inhibiton zones test from Ethyl Caffeate (EC) against some bacteria. A.) Inhibition zone of EC (5, 10, 25, and 50 µg/ml) against *V. parahaemolyticus*. B.) Inhibition zone of EC (5, 10, 25, and 50 µg/ml) against *Lact. plantarum*, *L. acidophilus*, *L. reuteri*, and *P. acidilactici*.

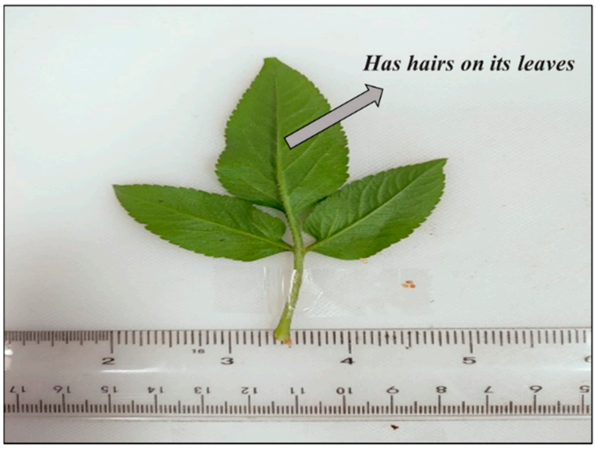

Bidens pilosa

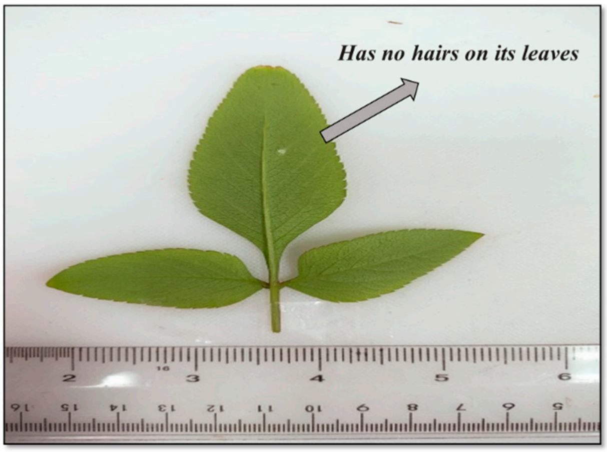

Bidens alba

Figure S5. The difference between Bidens pilosa and Bidens alba can be identify based on the hairs on its leaves. The distinction based on Bartolome et al., 2013 [13] .
